# Impact of the occurrence of a response shift on the determination of the minimal important difference in a health-related quality of life score over time

**DOI:** 10.1186/s12955-016-0569-5

**Published:** 2016-12-03

**Authors:** Ahmad Ousmen, Thierry Conroy, Francis Guillemin, Michel Velten, Damien Jolly, Mariette Mercier, Sylvain Causeret, Jean Cuisenier, Olivier Graesslin, Zeinab Hamidou, Franck Bonnetain, Amélie Anota

**Affiliations:** 1 Methodology and Quality of Life Unit in Oncology, University Hospital of Besançon, Besançon, France; 2 INSERM UMR 1098, University of Franche-Comte, Besançon, France; 3 Medical Oncology Department, Centre Alexis Vautrin, Nancy, France; 4 French National Platform Quality of Life and Cancer, Besançon, France; 5 Inserm CIC-EC 1433, and CHU, Clinical Epidemiology and Evaluation Department, Nancy, France; 6 Department of Epidemiology and Public Health, Faculty of Medicine, EA 3430, University of Strasbourg, Strasbourg, France; 7 Pôle Recherche – Innovations, University Hospital of Reims, Reims, France; 8 Surgery Department, Centre Georges François Leclerc, Dijon, France; 9 Gynecological and Obstetric Department, Institut Mère Enfant, University Hospital of Reims, Reims, France; 10 Public health laboratory, EA 3279, Aix-Marseille University, Marseille, France

**Keywords:** Health-related quality of life, Response shift, Minimal important difference, Then-test, Anchor-based method

## Abstract

**Background:**

An important challenge of the longitudinal analysis of health-related quality of life (HRQOL) is the potential occurrence of a Response Shift (RS) effect. While the impact of RS effect on the longitudinal analysis of HRQOL has already been studied, few studies have been conducted on its impact on the determination of the Minimal Important Difference (MID). This study aims to investigate the impact of the RS effect on the determination of the MID over time for each scale of both EORTC QLQ-C30 and QLQ-BR23 questionnaires in breast cancer patients.

**Methods:**

Patients with breast cancer completed the EORTC QLQ-C30 and the EORTC QLQ-BR23 questionnaires at baseline (time of diagnosis; T0), three months (T1) and six months after surgery (T2). Four hospitals and care centers participated in this study: cancer centers of Dijon and Nancy, the university hospitals of Reims and Strasbourg At T1 and T2, patients were asked to evaluate their HRQOL change during the last 3 months using the Jaeschke transition question. They were also asked to assess retrospectively their HRQOL level of three months ago.

The occurrence of the RS effect was explored using the then-test method and its impact on the determination of the MID by using the Anchor-based method.

**Results:**

Between February 2006 and February 2008, 381 patients were included of mean age 58 years old (SD = 11). For patients who reported a deterioration of their HRQOL level at each follow-up, an increase of RS effect has been detected between T1 and T2 in 13/15 dimensions of QLQ-C30 questionnaire, and 4/7 dimensions of QLQ-BR23 questionnaire. In contrast, a decrease of the RS effect was observed in 8/15 dimensions of QLQ-C30 questionnaire and in 5/7 dimensions of QLQ-BR23 questionnaire in case of improvement. At T2, the MID became ≥ 5 points when taking into account the RS effect in 10/15 dimensions of QLQ-C30 questionnaire and in 5/7 dimensions of QLQ-BR23 questionnaire.

**Conclusions:**

This study highlights that the RS effect increases over time in case of deterioration and decreases in case of improvement. Moreover, taking the RS into account produces a reliable and significant MID.

**Electronic supplementary material:**

The online version of this article (doi:10.1186/s12955-016-0569-5) contains supplementary material, which is available to authorized users.

## Background

In oncology clinical trials including health-related quality of life (HRQOL) as an endpoint, the main objective is to assess the impact of the treatment on patient’s HRQOL level over time. Consequently, a longitudinal assessment of HRQOL is desirable. The interpretation of the results of the longitudinal analysis of such data must be made in both statistical and clinical point of view in order to produce meaningful results for both patients and clinicians [1, 2]. The minimal important difference (MID) was defined as the smallest change between two scores in a treatment outcome that a patient would identify as important [3–6].

For the European Organization of Research and Treatment of Cancer (EORTC) HRQOL questionnaires, the MID is generally fixed to 5 or 10 points for each score standardized on a 0–100 scale [2]. Nevertheless, this MID must be studied and determined for each HRQOL questionnaire and according to each cancer site. This was already made for the EORTC module of lung and brain cancer as example [7, 8]. To our knowledge, it has not yet been done for the EORTC QLQ-BR23 module for breast cancer patients. Furthermore, it is mandatory to not ignore the importance of this MID and to take it into account in the interpretation of HRQOL results. Indeed, the longitudinal analysis of HRQOL remains complex, particularly due to the potential occurrence of a response shift (RS) effect characterizing the process of adaptation of the patient in relation to the illness and its treatment [9, 10]. Thus, patients may not assess with the same criteria their HRQOL level over time.

The RS refers to a change in the meaning of HRQOL over time. Its definition proposed by Sprangers and Schwartz consists of three components:

- A recalibration: change in the respondent’s internal standards of measurement;
- A reprioritization: change in the importance of the component domains that constitute HRQOL;
- A reconceptualization: redefinition of the concept of HRQOL [9, 10].

Several methodological or statistical methods have been proposed to characterize the occurrence of the RS effect, such as the then-test [9] or structural equation modeling [11]. The then-test consists to ask to patients post-treatment to provide their current levels (post-test) but also their pre-test levels in retrospect (then-test). Its impact on longitudinal HRQOL analysis has also been studied in breast cancer patients [12]. However, at this time, few researches have focused on the impact of RS effect on the determination of the MID [13]. Thus, the MID recommended for future studies could thus be under or over estimated by a potential RS effect.

The occurrence of the RS effect could affect the interpretation of change in HRQOL scores. In this case, we need to assess the occurrence of the RS to obtain a valid and reliable assessment of change over time. In particular, when we have longitudinal data to determine the MID, it is therefore important to take into account this RS to assess the true change represented by the MID.

Many studies aimed to estimate the MID without taking into account the occurrence of the RS [3, 4]. To our knowledge, only one study explored the impact of the RS on the MID determination. This study demonstrated that the recalibration component of the RS effect does not have an important effect in patients with multiple myeloma who respond to treatment, i.e. for which a HRQOL improvement was observed. However, the author showed that RS does have an important effect in case of deterioration of patient’s HRQOL level [13]. Thus it seems to be essential to study the impact of the RS effect in studies aiming to determine the MID, and to study if this RS impact differentially patients who improved to those who deteriorate over time. Only two measurement times (at inclusion and after three months) were considered in the study of Kvam et al. which allow detecting the importance of RS effect on deteriorating or improving of HRQOL and its direction after three months [13]. However, since the RS effect is a longitudinal process, it could be relevant to include more time points in order to study the longitudinal change of the RS effect over time.

In this context, the objective of this work was to study the impact of the recalibration component of the RS effect on the determination of the MID in breast cancer patients between three measurement times using the EORTC QLQ-C30 cancer specific questionnaire and its breast cancer module QLQ-BR23.

## Methods

### Patients

Data from a prospective, multicenter, cohort study were used including all women hospitalized for the diagnosis or treatment of primary breast cancer or for a suspicion of breast cancer. Patients who have other primary cancer sites were excluded. Patients already hospitalized or treated for breast cancer were not included. Written informed consent was obtained from all participants. The protocol was approved by the ethics committees (“Comité de Protection des Personnes”). The complete design of this study was extensively described elsewhere [14].

### Study design

#### HRQOL questionnaires

HRQOL was assessed using the EORTC QLQ-C30 cancer-specific questionnaire and its QLQ-BR23 breast cancer module. Three measurement times were used: at baseline (initial examination or initial hospitalization, T0), three months (T1) and six months later (T2).

The QLQ-C30 consists of 30 items measuring five functional scales (physical, role, emotional, cognitive and social functioning), a global health status (GHS), financial difficulties and eight scales of symptoms (fatigue, nausea and vomiting, pain, dyspnea, insomnia, appetite loss, constipation, diarrhea) [15]. One score is generated per dimension and standardized on a 0 to 100 scale in order that a high score reflects a high GHS, functional and symptomatic level [16].

The QLQ-BR23 module is specific to breast cancer. It includes 23 items allowing to assess four functional scales (body image, sexual functioning, sexual enjoyment, future perspectives) and four symptom scales (systemic therapy side effects, breast symptoms, arm symptoms, upset by hair loss) [17]. As for the QLQ-C30, one score is generated per dimension on a 0–100 scale in order that a high score represents a high level of functioning and a high symptomatic level.

Questionnaires have been distributed by a clinical research assistant to the patients during the hospitalization or after a consultation or sending by the post.

### Then-test assessment

In this study, the then-test method was used to detect changes in internal standards, namely the “recalibration” component of the RS [18].

At each follow-up time point, one prospective and one retrospective measurement were performed. For the retrospective measurement (then-test) at T1, patients were asked to re-evaluate their baseline HRQOL level (three months before). At T2 (six months), patients were asked to re-evaluate their HRQOL level at three months (retrospective assessment of HRQOL level at T1 (three months)).

### Assessment of change in HRQOL level

The anchor-based method was used to determine the MID according to the Jaeschke transition question [3]. At three (T1) and six months (T2), patients were asked to evaluate their HRQOL change in the last three months. The question was asked by the following way:

“During the past three months, do you consider your HRQOL:

- Did not change globally
- Deteriorated: very much, much, a little
- Improved: a little, much, very much”

Since the Jaeschke transition question was asked to the patients at T1 and then at T2, patients can deteriorate between T0 and T1 and then can improve between T1 and T2. Thus, patients in the group “little worse” at T1 can then be in the group “little better” at T2.

To facilitate the interpretation of the results and to yield sufficient numbers of patients in each category, we brought the two categories “very much” and “much” in a single category to get finally five response categories for the anchor (much better, little better, unchanged, little worse, and much worse).

### Statistical methods

#### Statistical considerations and missing data

All dimensions of both QLQ-C30 and QLQ-BR23 questionnaires were analysed except the hair loss dimension of the QLQ-BR23 due to missing data (few patients concerned at the stage of the beginning of the treatment).

Only patients with available data at each time measurement were included in the analyses.

All tests were performed at the statistical level of 0.05 with no adjustment on multiple tests. All tests were performed at the statistical level of 0.05 with no adjustment on multiple tests. All *p*-values were given for information only since sample sizes for each test do not allow to produce some results with a high statistical power. The analyses and tests were made as an exploratory purpose only, we are more interested about clinical meaning of the difference instead of statistical significance.

Scores were calculated according to the recommendations of the EORTC scoring manual [15]: if at least half of the items per dimension were answered, the score was estimated on available items, i.e. considering that missing items were equal to the mean of answered items (simple imputation by the personal mean).

Missing data profile was already explored in a previous study [19]. They were considered as missing at random.

### Descriptive analysis

Baseline sociodemographics and clinical characteristics of the patients as well as baseline HRQOL scores were described using mean and standard deviation (SD) for continuous variables and frequencies with percentages for qualitative variables.

### Detection of the recalibration effect of the RS

RS analyses were performed on patients with available scores at both the the then-test and the corresponding pre-test. The mean differences between the prospective measure performed at T0 and the retrospective measurement performed at T1 as well as between the prospective measure performed at T1 and the retrospective measurement performed at T2 were calculated for each HRQOL score. Results were presented according to each response category of the anchor’s question in order to detect the magnitude of the RS effect according to the observed changes.

As the MID is defined as the smallest change between two scores, we were particularly interested in the two categories “little worse” and “little better” to interpret the results.

We looked at the direction of the RS effect: a positive (respectively, negative) value of RS indicates that patients had overestimated (respectively, underestimated) their HRQOL level, their functional or symptomatic level at the previous measurement time.

Then, we were interested in the evolution of the magnitude of the RS effect over time, it means, if the RS effect had increased or decreased over time in absolute value.

Finally, the direction of the response shift effect over time was also analyzed, i.e. if the RS remained positive or negative at both follow-up time points.

The impact of sample size was indicated by calculation of the 95% confidence intervals (95%CI).

The *p*-values calculated by the non-parametric Wilcoxon paired test were also presented to indicate the statistical significance of the RS.

The effect size (ES) was calculated to detect the magnitude of the recalibration component of the RS effect for each category of the anchor. The ES represents the mean change between the pre-test and the then-test divided by the standard deviation (SD) of the pre-test score. We used Cohen’s generally accepted criteria for interpreting the magnitude of the ES in absolute value: an ES of at least 0.20 was considered as a small change, between 0.2 and 0.50 as a moderate change, and greater than 0.80 as an important change [20].

### Observed and adjusted MID

MID were determined by calculating the observed changes (i.e. without taking into account the RS effect) and the adjusted changes (i.e. taking into account the RS effect) given by post-test minus pre-test and post-test minus then-test respectively. Observed changes for each score were estimated on patients with the corresponding score available at both the post-test and pre-test measurement times. Adjusted changes for each score were performed on patients with the corresponding score available for both the post-test and then-test. In the both cases, the mean differences were calculated for each HRQOL scores according to each response category of the anchor’s question.

The impact of sample size was indicated by calculation of the 95%CI of the mean difference.

The global range for the observed and adjusted MID for all dimensions was then reported by questionnaire and measurement times.

Results of the observed and adjusted MID were finally compared to the threshold of 5 points MID which is widely used for the EORTC HRQOL questionnaires [2].

All analyses were performed using R statistical software (version 3.2.1) [21].

## Results

### Patients

**Fig. 1:**
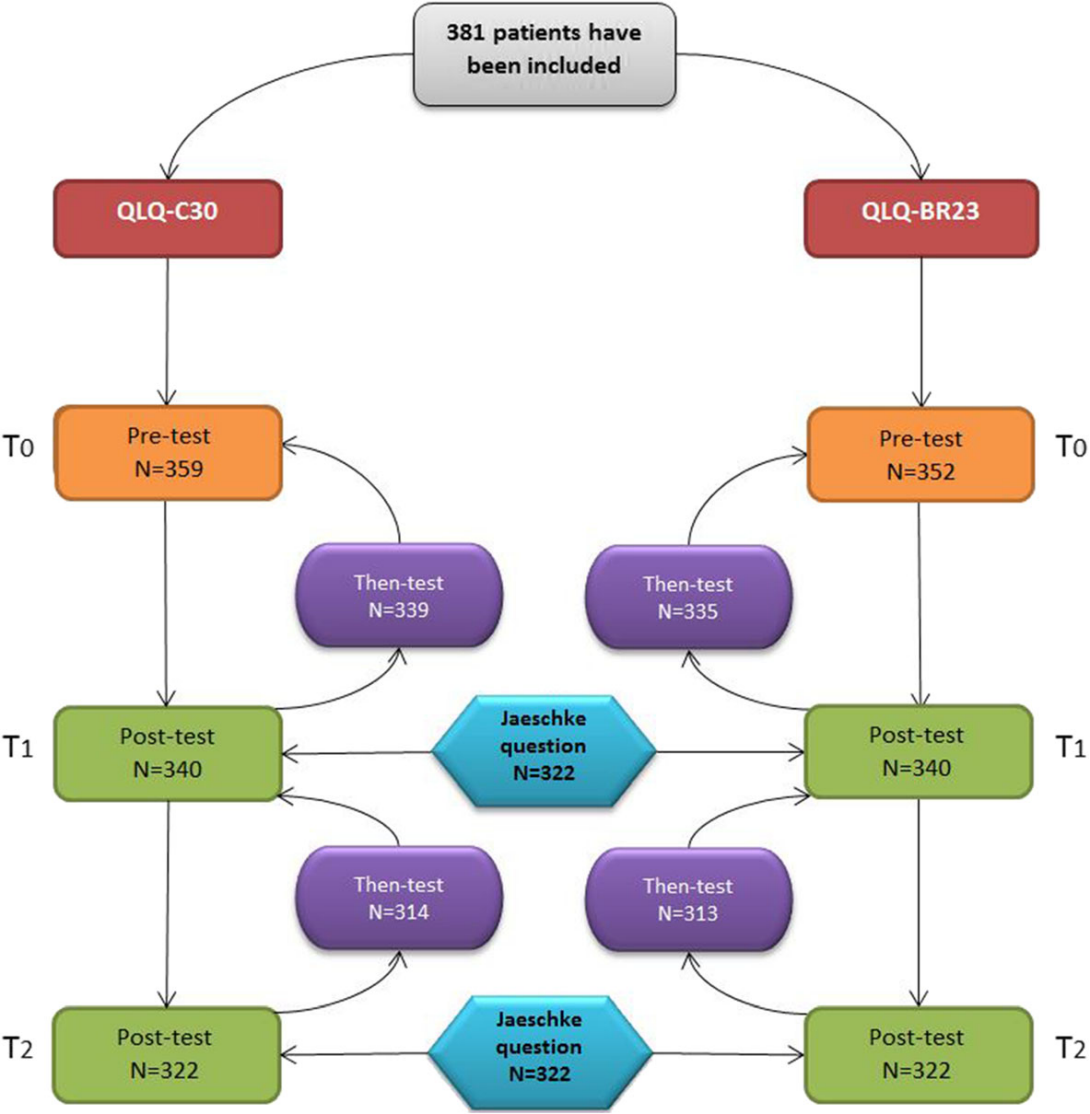
Flowchart with number of questionnaires collected at each measurement time

Between February 2006 and February 2008, 381 patients with confirmed or suspicion breast cancer were included in the four participating centers (Fig. 1). A difference between centers was observed in terms of questionnaires completions rate due to logistics problems. Mean age was 58.4 (SD = 11) years. Three hundred and forty (89.2%) patients had a confirmed breast cancer. the clinical and socio- demographic characteristics of all patients were described in Table 1.

**Table 1:** Patients characteristics

	Number	Percent
Hospital		
Dijon	271	71.1
Nancy	74	19.4
Reims	18	4.7
Strasbourg	18	4.7
Inclusion criteria		
Confirmed primary breast cancer	242	63.5
Suspicion of primary breast cancer	138	36.2
Unknown	1	0.3
Cancer		
Confirmed	337	89.2
Not confirmed	41	10.0
Unknown	3	0.8
Stage (AJCC)		
0	78	20.5
1	135	35.4
2	118	31
3 or 4	15	4
Unknown	35	9.1
Lymph node dissection (LND)		
Axillary LND	138	36.2
Sentinel lymph node biopsy	131	34.4
ALND + SLNB	32	8.4
No LND	75	19.7
Unknown	5	1.3
Surgery type		
Mastectomy	124	32.6
No mastectomy	241	63.3
Unknown	16	4.2
Chemotherapy		
Yes	155	40.7
No	218	57.2
Unknown	8	2.1
Radiotherapy		
Yes	254	66.7
No	119	31.2
Unknown	8	2.1
Hormone therapy		
Yes	170	44.6
No	203	53.3
Unknown	8	2.1

### Detection of the recalibration component of the Response shift effect

Tables 2 and 3 present the results of RS effect at T1 and T2 for the QLQ-C30 and QLQ-BR23 questionnaires respectively (see Additional file 1: Tables S1 and S2 for complementary results).

**Table 2:** The Response shift effect for the QLQ-C30 questionnaire after three and six months for all patients and three main categories of the anchor

QLQ-C30	After 3 months					After 6 months				
	Between T0 and T1					Between T1 and T2				
	N	RS (SD)	95%CI	*P*-value	ES	N	RS (SD)	95% CI	*P*-value	ES
GHS	251	3.82 (17.85)		0.02	0.20	251	−0.03 (21.04)		0.97	0
Little worse	71	3.87 (15.86)	(1, 7)	0.24	0.25	51	−8.33 (19.22)	(−13, −4)	0.03	−0.42
No change	43	6.78 (15.67)	(3, 11)	0.08	0.40	50	8 (19.04)	(3, 13)	0.03	0.42
Little better	58	1.29 (17.51)	(−3, 5)	0.67	0.08	67	−0.25 (21.61)	(−5, 4)	0.80	−0.01
Physical functioning	255	1.54 (12.95)		0.30	0.11	255	−5.2 (12.86)		0	−0.32
Little worse	73	−0.53 (11.11)	(−3, 2)	0.49	−0.05	53	−9.56 (10.42)	(−12, −7)	0	−0.52
No change	46	1.30 (13.67)	(−2, 5)	0.17	0.09	51	−2.29 (8.5)	(−4, 0)	0.13	−0.26
Little better	57	3.83 (14.47)	(1, 7)	0.45	0.28	69	−3.94 (14.71)	(−7, −1)	0.04	−0.25
Role functioning	253	6.06 (21.71)		0	0.32	253	−8.17 (27.07)		0	−0.29
Little worse	73	6.62 (19.98)	(3, 11)	0.04	0.46	51	−15.69 (28.95)	(−22, −9)	0	−0.52
No change	46	3.26 (16.34)	(−1, 7)	0.07	0.19	51	−1.96 (18.45)	(−6, 2)	0.67	−0.11
Little better	55	10 (26.57)	(4, 16)	0.05	0.45	68	−5.88 (26.67)	(−11, 0)	0.24	−0.21
Emotional functioning	254	−7.56 (21.03)		0	−0.29	254	2.20 (22.68)		0.20	0.09
Little worse	72	−9.34 (20.97)	(−13, −5)	0	−0.38	52	−5.66 (19.77)	(−10, −1)	0.16	−0.24
No change	44	−8.52 (17.14)	(−13, −4)	0.03	−0.42	50	8.94 (21.58)	(4, 14)	0.04	0.47
Little better	59	−11.11 (23.15)	(−16, −6)	0.01	−0.43	69	5.68 (21.83)	(1, 10)	0.10	0.23
Cognitive functioning	254	−3.94 (16.85)		0.01	−0.19	254	−3.08 (20.03)		0.04	−0.15
Little worse	73	−5.48 (20.04)	(−9, −2)	0.08	−0.26	52	−10.58 (21.9)	(−16, −5)	0.02	−0.42
No change	44	−1.52 (10.67)	(−4, 1)	0.69	−0.11	50	−2.33 (16.5)	(−6, 2)	0.35	−0.16
Little better	57	−5.85 (19.29)	(−10, −2)	0.08	−0.27	69	0.97 (18.28)	(−3, 5)	0.98	0.05
Social functioning	249	5.09 (19.43)		0	0.28	249	−5.96 (24.47)		0.01	−0.23
Little worse	72	5.32 (19.94)	(1, 9)	0.05	0.39	52	−10.90 (25.54)	(−17, −5)	0.02	−0.40
No change	42	3.17 (14.37)	(−1, 7)	0.08	0.24	49	−4.76 (16.67)	(−9, −1)	0.40	−0.22
Little better	59	3.67 (20.55)	(−1, 8)	0.59	0.18	68	−1.23 (24.32)	(−6, 4)	0.91	−0.05
Financial difficulties	240	−1.11 (15.32)		0.30	−0.08	240	2.50 (16.26)		0.22	0.12
Little worse	69	−1.45 (12.04)	(−4, 1)	0.58	−0.14	49	8.16 (24.09)	(2, 14)	0.13	0.30
No change	41	−1.63 (7.27)	(−4, 0)	0.31	−0.31	47	−0.71 (4.86)	(−2, 0)	0.57	−0.15
Little better	58	0.57 (15.91)	(−3, 4)	0.86	0.03	66	−1.01 (16.51)	(−4, 2)	0.82	−0.05
Fatigue	254	−1.20 (19.42)		0.51	−0.06	254	10.83 (23.93)		0	0.41
Little worse	73	−4.57 (21.36)	(−9, 0)	0.31	−0.33	53	19.92 (23.23)	(15, 25)	0	0.85
No change	46	−2.17 (15.29)	(−6, 2)	0.62	−0.12	51	3.70 (20.08)	(−1, 8)	0.32	0.20
Little better	57	−4.58 (21.29)	(−9, 0)	0.35	−0.23	68	9.97 (23.76)	(5, 15)	0.01	0.44
Nausea and vomiting	255	−1.37 (13.59)		0.26	−0.13	255	3.59 (17.65)		0	0.19
Little worse	74	−0.45 (15.35)	(−3, 3)	0.71	−0.05	53	4.09 (14.58)	(1, 7)	0.09	0.26
No change	46	0.72 (9.91)	(−2, 3)	1	0.04	51	0.98 (10.23)	(−1, 3)	0.10	0.10
Little better	57	−5.26 (15.16)	(−9, −2)	0.05	−0.87	70	6.67 (25.44)	(2, 12)	0.05	29
Pain	260	−3.33 (22.51)		0.14	−0.16	260	5.83 (22.2)		0	0.23
Little worse	75	−4.67 (21.32)	(−9, −1)	0.29	−0.24	54	15.43 (26.27)	(9, 21)	0	0.56
No change	46	1.45 (18.2)	(−3, 6)	0.83	0.09	52	2.24 (13.21)	(−1, 5)	0.30	0.18
Little better	57	−7.31 (23.57)	(−13, −2)	0.16	−0.35	70	−0.24 (23.14)	(−5, 4)	0.80	−0.01
Dyspnea	251	1.20 (15.16)		0.59	0.06	251	3.98 (22.4)		0.03	0.17
Little worse	73	2.28 (15.04)	(−1, 5)	0.38	0.11	52	3.85 (27.74)	(−3, 10)	0.14	0.17
No change	45	0 (12.31)	(−3, 3)	0.77	0	51	0.65 (10.52)	(−2, 3)	0.79	0.05
Little better	55	0.61 (17.56)	(−3, 5)	0.77	0.03	67	3.98 (22.11)	(−1, 8)	0.51	0.17
Insomnia	249	6.83 (30.64)		0.01	0.22	249	3.08 (30.41)		0.30	0.10
Little worse	69	2.42 (29.33)	(−3, 8)	0.49	0.08	52	10.9 (37.18)	(2, 2)	0.07	0.33
No change	46	7.25 (26.21)	(1, 14)	0.27	0.25	50	3.33 (25.42)	(−3, 9)	0.24	0.18
Little better	55	7.88 (37.93)	(−1, 16)	0.11	0.27	67	−1.49 (29.83)	(−8, 5)	0.74	−0.05
Appetite loss	249	2.28 (21.05)		0.23	0.11	249	4.69 (22.2)		0.03	0.19
Little worse	73	0.91 (20.77)	(−3, 5)	0.55	0.06	53	13.84 (24.84)	(8, 20)	0	0.51
No change	46	4.35 (13.35)	(1, 8)	0.19	0.22	50	0.67 (15.78)	(−3, 4)	0.98	0.03
Little better	54	1.85 (22.82)	(−3, 7)	0.79	0.09	68	1.96 (21.46)	(−2, 6)	0.74	0.09
Constipation	247	0 (22.61)		0.87	0	247	4.18 (25.89)		0.05	0.16
Little worse	68	−4.90 (18.45)	(−9, −1)	0.19	−0.26	48	4.17 (25.38)	(−2, 10)	0.29	0.16
No change	42	2.38 (18.61)	(−2, 7)	0.42	0.11	48	2.08 (14.43)	(−1, 6)	0.43	0.15
Little better	58	0 (27.22)	(−6, 6)	0.87	0	70	2.86 (35.32)	(−4, 10)	0.71	0.10
Diarrhea	248	2.42 (14.99)		0.09	0.15	248	0.94 (20.28)		0.42	0.06
Little worse	71	3.29 (16.09)	(0, 6)	0.28	0.18	48	3.47 (14.16)	(0, 7)	0.22	0.23
No change	43	1.55 (12.50)	(−2, 5)	0.54	0.13	48	−0.69 (14.57)	(−4, 3)	0.98	−0.07
Little better	56	2.98 (17.15)	(−1, 7)	0.34	0.16	69	0 (26.81)	(−5, 5)	0.86	0

*GHS* global health status, *RS* response shift, *SD* standard deviation, *CI* confidence interval, *ES* effect size

**Table 3:** The Response shift effect for the QLQ-BR23 questionnaire after three and six months for all patients and three main categories of the anchor

	After 3 months					After 6 months				
	Between T0 and T1					Between T1 and T2				
QLQ-BR23	N	RS (SD)	95%CI	*P*-value	ES	N	RS (SD)	95%CI	*P*-value	ES
Body image	232	6.50 (20.73)		0.01	0.37	232	−7.18 (22.33)		0.01	−0.23
Little worse	70	5.04 (17.89)	(1, 9)	0.11	0.32	47	−11.17 (21.1)	(−16, −6)	0.03	−0.37
No change	41	3.25 (14.54)	(−1, 7)	0.11	0.20	46	−3.62 (12.25)	(−7, −1)	0.28	−0.19
Little better	49	9.98 (23)	(4, 15)	0.11	0.62	65	−2.74 (24.7)	(−8, 2)	0.63	−0.09
Sexual functioning	198	0.76 (15.06)		0.77	0.03	198	4.29 (18.88)		0.10	0.19
Little worse	56	0.60 (15.56)	(−3, 4)	0.90	0.02	38	10.96 (19.86)	(6, 16)	0.07	0.52
No change	34	1.96 (15.77)	(−3, 7)	0.73	0.08	41	0.41 (18.44)	(−4, 5)	0.90	0.01
Little better	43	−1.94 (12.71)	(−5, 1)	0.77	−0.08	56	0.89 (16.02)	(−3, 4)	0.88	0.05
Sexual enjoyment	74	−4.05 (20.74)		0.26	−0.16	74	3.15 (23.49)		0.21	0.13
Little worse	23	−2.90 (24.44)	(−12, 6)	0.59	−0.16	16	2.08 (30.96)	(−11, 16)	0.55	0.10
No change	16	−6.25 (13.44)	(−12, 0)	0.51	−0.23	23	1.45 (25.58)	(−8, 11)	0.69	0.06
Little better	11	−3.03 (23.35)	(−16, 10)	0.80	−0.10	16	2.08 (14.75)	(−4, 9)	0.83	0.08
Future perspectives	236	−7.91 (30.3)		0.01	−0.27	236	1.27 (30.77)		0.64	0.04
Little worse	68	−16.18 (27.31)	(−22, −11)	0	−0.56	48	−4.17 (31.98)	(−12, 4)	0.49	−0.13
No change	43	−13.18 (29.22)	(−21, −6)	0.04	−0.47	49	4.76 (34.69)	(−4, 13)	0.47	0.18
Little better	48	−6.94 (32.95)	(−15, 1)	0.22	−0.28	63	7.41 (25.71)	(2, 13)	0.18	0.23
Systemic therapy side effects	252	0.42 (12.32)		0.73	0.03	252	8.16 (16.76)		0	0.42
Little worse	72	0.96 (12.49)	(−1, 3)	0.78	0.07	53	13.76 (19.15)	(9, 18)	0	0.73
No change	45	−0.56 (8.12)	(−3, 1)	0.90	−0.04	52	2.22 (8.64)	(0, 4)	0.53	0.19
Little better	54	0 (12.64)	(−3, 3)	0.47	0	65	8.13 (19.39)	(4, 12)	0	0.45
Breast symptoms	212	−2.59 (19.13)		0.69	−0.18	212	8.54 (20.19)		0	0.35
Little worse	57	4 (17.94)	(0, 8)	0.09	0.24	47	10.76 (19.94)	(6, 16)	0.01	0.4
No change	36	−0.62 (11.35)	(−4, 3)	0.68	−0.07	42	7.34 (16.22)	(3, 12)	0.02	0.47
Little better	53	−9.49 (21.49)	(−14, −5)	0.12	−0.76	59	5.84 (14.8)	(3, 9)	0.17	0.23
Arm symptoms	235	−1.94 (16.33)		0.50	−0.14	235	3.81 (19.17)		0.02	0.21
Little worse	67	0.66 (18)	(−3, 4)	0.55	0.05	48	10.19 (20.06)	(5, 15)	0	0.47
No change	41	1.36 (7.93)	(−1, 3)	0.31	0.16	50	4 (10.88)	(1, 7)	0.01	0.36
Little better	55	−3.13 (18.07)	(−7, 1)	0.45	−0.18	64	0 (17.51)	(−4, 4)	0.71	0

*RS* response shift, *SD* standard deviation, *CI* confidence interval, *ES* effect size

For 18 over 22 dimensions analysed of both questionnaires, an increase of the magnitude of the RS effect was observed in case of little deterioration over time, i.e. between T0 and T1 and then between T1 and T2 (i.e. category “little worse” for the anchor at each follow-up time point).

To illustrate:

- For the insomnia dimension of the QLQ-C30 questionnaire, the RS effect was equal to 2.42 in mean at three months reflecting that patients had overestimated their baseline insomnia level, considering the retrospective measure at three months as the reference. The RS effect became more important after six months by increasing to 10.9 in mean reflecting an overestimation of the insomnia level at T1. Thus, the magnitude of the RS effect increased at six months as compared to three months, with a positive direction of the RS at both time points.
- Regarding the body image dimension of the QLQ-BR23 module, the magnitude of the mean RS effect also increase between each follow-up time point from 5.04 to 11.17 in absolute value but with an opposite direction of RS was shown at T1 equal to 5.04 in mean compared with its value after six months by increasing to −11.17 with underestimation of body image pre-test score at T1.

ES indicate a moderate RS effect in case of deterioration after six months for physical, role, cognitive social and sexual functioning as well as for pain, appetite loss, and systemic therapy side effects dimensions (ES > 0.5) and an important RS effect for fatigue (ES > 0.8). Furthermore, a statistically significance of RS effect has been indicated (for information only since sample sizes for each test do not allow to produce some results with a high statistical power) for 12 dimensions of both QLQ-C30 and QLQ-BR23 questionnaires in case of deterioration after six months (*P*-value < 0.05).

A decrease of the magnitude of the RS effect was observed for 13/22 dimensions analyzed in case of improvement betweenT1 and T2 (i.e. category “little better” for the anchor at T1 and at T2). For example:

- A decrease of RS effect with underestimation of pain level at T0 and T1 from −7.31 after three months to −0.24 after six months for;
- A decrease of RS effect from 9.98 with overestimation of baseline body image level to −2.74 with underestimation of body image pre-test score at T1.

For all dimensions for which a decrease of RS effect was observed between T1 and T2 in case of improvement of HRQOL level, the ES indicated an insignificant RS (ES ≤ 0.2) or a small change (0.2 < ES ≤ 0.5). In addition, results were not statistically significant (*p*-values of the Wilcoxon test ≥ 0.05).

In case of deterioration, the same direction of RS between T1 and T2 was observed for 11/22 dimensions of both QLQ-C30 and QLQ-BR23 questionnaires. In case of improvement, the direction of the RS effect remained the same for only 3/22 dimensions (namely, pain, dyspnea and appetite loss dimensions).

### Observed and adjusted MID

Tables 4 and 5 represent the results of the observed and adjusted MID at 3 and 6 months for the QLQ-C30 and QLQ-BR23 respectively (see Additional file 1: Tables S3 and S4 for complementary results).

**Table 4:** Observed and adjusted changes of the QLQ–C30 questionnaire after three and six months

QLQ-C30	After 3 months					After 6 months				
	Between T0 and T1					Between T1 and T2				
		Observed changes		Adjusted changes			Observed changes		Adjusted changes	
		(post-test - pre-test)		(post-test - then-test)			(post-test - pre-test)		(post-test - then-test)	
	N	Mean (SD)	95% CI	Mean (SD)	95% CI	N	Mean (SD)	95% CI	Mean (SD)	95% CI
GHS	251					251				
Little worse	71	−16.31 (16.99)	(−20, −13)	−12.44 (16.78)	(−16, −9)	51	0.49 (15.4)	(−3, 4)	−7.84 (15.22)	(−11, −4)
No change	43	−2.13 (16.17)	(−6, 2)	4.65 (17.85)	(0, 9)	50	−1 (16.12)	(−5, 3)	7 (14.71)	(4, 10)
Little better	58	0.29 (18.4)	(−4, 4)	1.58 (14.93)	(−2, 5)	67	7.21 (17.22)	(4, 11)	6.97 (16.70)	(4, 10)
Physical functioning	255					255				
Little worse	73	−11.76 (10.99)	(−14, −10)	−12.28 (12.71)	(−15, −10)	53	0.38 (11.43)	(−2, 3)	−9.18 (12.83)	(−12, −6)
No change	46	−3.99 (10.17)	(−7, −1)	-2.68 (12.76)	(−6, 0)	51	0.72 (6.97)	(−1, 2)	−1.57 (6.34)	(−3, 0)
Little better	57	−4.53 (12.89)	(−7, −2)	-0.70 (11.86)	(−3, 2)	69	0.85 (13.02)	(−2, 3)	−3.09 (14.03)	(−6, 0)
Role functioning	253					253				
Little worse	73	−25.57 (21.71)	(−30, −21)	-18.95 (23.78)	(−24, −14)	51	−1.96 (29.18)	(−9, 5)	−17.65 (29.14)	(−24, −11)
No change	46	−2.90 (19.66)	(−8, 2)	0.36 (21.80)	(−5, 6)	51	3.92 (15.49)	(0, 8)	1.96 (19.05)	(−3, 6)
Little better	55	−9.09 (20.74)	(−14, −4)	0.91 (24.72)	(−5, 6)	68	5.88 (21.89)	(1, 1)	0 (23.92)	(−5,5)
Emotional functioning	254					254				
Little worse	72	3.05 (24.09)	(−2, 8)	-6.29 (22.02)	(−11, −2)	52	−0.53 (20.19)	(−5, 4)	−6.20 (16.43)	(−10, −2)
No change	44	19.44 (18.31)	(15, 24)	10.92 (18.26)	(6, 16)	50	−0.78 (15.55)	(−4, 3)	8.17 (21.26)	(3, 13)
Little better	59	15.07 (26.33)	(9, 21)	3.95 (19.51)	(0, 8)	69	1.53 (20.05)	(−2, 6)	7.21 (19.13)	(3, 11)
Cognitive functioning	254					254				
Little worse	73	−5.48 (19.46)	(−9, −2)	-10.96 (18.26)	(−15, −7)	52	0.96 (17.9)	(−3, 5)	−9.62 (19.06)	(−14, −5)
No change	44	0.76 (13.43)	(−3, 4)	-0.76 (12.43)	(−4, 2)	50	2.67 (12.31)	(0, 6)	0.33 (15.97)	(−3, 4)
Little better	57	5.26 (21.4)	(1, 1)	-0.58 (17.24)	(−4, 3)	69	3.62 (13.37)	(1, 6)	4.59 (17.82)	(1, 8)
Social functioning	249					249				
Little worse	72	−20.37 (21.34)	(−25, −16)	-15.05 (14.84)	(−18, −12)	52	0.64 (24.47)	(−5, 6)	−10.26 (20.39)	(−15, −6)
No change	42	−3.97 (21.08)	(−9, 2)	-0.79 (17.25)	(−5, 4)	49	4.42 (19.18)	(0, 9)	−0.34 (12.50)	(−3, 3)
Little better	59	−6.78 (19.6)	(−11, −3)	-3.11 (18.95)	(−7, 1)	68	3.43 (20.68)	(−1, 8)	2.21 (21.34)	(−2, 7)
Fatigue	254					254				
Little worse	73	27.32 (21.87)	(23, 32)	22.75 (22.38)	(18, 27)	53	0.21 (16.34)	(−4, 4)	20.13 (24.17)	(15, 26)
No change	46	7.73 (18.42)	(3, 12)	5.56 (19.42)	(1, 10)	51	−1.53 (19.5)	(−6, 3)	2.18 (19.75)	(−2, 7)
Little better	57	7.89 (19.47)	(4, 12)	3.31 (17.97)	(−1, 7)	68	−7.68 (18.24)	(−11, −4)	2.29 (22.92)	(−2, 7)
Nausea and vomiting	255					255				
Little worse	74	8.78 (19.35)	(5, 13)	8.33 (15.67)	(5, 11)	53	−3.14 (21.7)	(−8, 2)	0.94 (17.73)	(−3, 5)
No change	46	−0.72 (12.65)	(−4, 2)	0 (8.61)	(−2, 2)	51	−2.29 (10.01)	(−5, 0)	−1.31 (12.40)	(−4, 2)
Little better	57	9.06 (21.38)	(4, 14)	3.80 (17.82)	(0, 8)	70	−6.67 (23.98)	(−11, −2)	0 (13.31)	(−3, 3)
Pain	260					260				
Little worse	75	13.78 (24.87)	(9, 19)	9.11 (23.14)	(5, 14)	54	1.85 (22.35)	(−3, 7)	17.28 (25.89)	(11, 23)
No change	46	3.99 (14.57)	(0, 8)	5.43 (17.23)	(1, 10)	52	3.21 (14.02)	(0, 6)	5.45 (17.06)	(1, 9)
Little better	57	9.94 (20.62)	(5, 15)	2.63 (21.08)	(−2, 7)	70	2.14 (20.05)	(−2, 6)	1.90 (22.97)	(−3, 6)
Dyspnea	251					251				
Little worse	73	8.68 (24.86)	(4, 14)	10.96 (23.61)	(6, 16)	52	3.85 (26.94)	(−2, 10)	7.69 (18.22)	(3, 12)
No change	45	0.74 (19.45)	(−4, 6)	0.74 (18.10)	(−4, 5)	51	−0.65 (12.45)	(−4, 2)	0 (11.55)	(−3, 3)
Little better	55	1.21 (19.21)	(−3, 6)	1.82 (16.25)	(−2, 5)	67	3.48 (22.57)	(−1, 8)	7.46 (23.08)	(3, 12)
Insomnia	249					249				
Little worse	69	5.80 (35.22)	(−1, 13)	8.21 (28.24)	(3, 14)	52	4.49 (30.98)	(−3, 12)	15.38 (24.22)	(10, 21)
No change	46	−10.14 (35.04)	(−19, −1)	−2.90 (25.17)	(−9, 3)	50	−1.33 (21.25)	(−6, 4)	2 (28.89)	(−5, 9)
Little better	55	−7.27 (34.95)	(−15, 1)	0.61 (25.25)	(−5, 6)	67	1 (30.13)	(−5, 7)	−0.50 (22.09)	(−5, 4)
Appetite loss	249					249				
Little worse	73	10.05 (28.16)	(5, 16)	10.96 (27.25)	(6, 16)	53	−10.06 (25.8)	(−16, −4)	3.77 (20.32)	(−1, 8)
No change	46	−2.90 (20.88)	(−8, 2)	1.45 (17.15)	(−3, 6)	50	−2.67 (9.13)	(−5, −1)	−2 (12.44)	(−5, 1)
Little better	54	1.85 (22.82)	(−3, 7)	3.70 (15.41)	(0, 7)	68	−2.45 (20.21)	(−7, 2)	−0.49 (14.67)	(−3, 2)
Constipation	247					247				
Little worse	68	15.20 (30.16)	(9, 21)	10.29 (27.77)	(5, 16)	48	−1.39 (24.75)	(−7, 5)	2.78 (21.56)	(−2, 8)
No change	42	0 (19.48)	(−5, 5)	2.38 (18.61)	(−2, 7)	48	−3.47 (12.38)	(−6, 0)	−1.39 (9.62)	(−4, 1)
Little better	58	0 (26.49)	(−6, 6)	0 (16.52)	(−4, 4)	70	−2.86 (30.95)	(−9, 3)	0 (26.62)	(−5, 5)
Diarrhea	248					248				
Little worse	71	0 (21.08)	(−4, 4)	3.29 (18.07)	(0, 6)	48	0.69 (14.57)	(−3, 4)	4.17 (13.09)	(1, 7)
No change	43	−2.33 (15.25)	(−6, 2)	-0.78 (11.47)	(−4, 2)	48	−1.39 (9.62)	(−4, 1)	−2.08 (10.67)	(−5, 0)
Little better	56	−0.6 (21.55)	(−5, 4)	2.38 (20.94)	(−2, 7)	69	−1.93 (17.97)	(−6, 2)	−1.93 (20.52)	(−6, 2)
Financial difficulties	240					240				
Little worse	69	9.66 (22.94)	(5, 14)	8.21 (22.44)	(4, 13)	49	−3.40 (20.69)	(−8, 2)	4.76 (21.52)	(0, 1)
No change	41	4.07 (13.32)	(1, 8)	2.44 (8.79)	(0, 5)	47	0 (0)	(NA)	−0.71 (4.86)	(−2, 0)
Little better	58	1.15 (22.48)	(−4, 6)	1.72 (15.82)	(−2, 5)	66	1.52 (9.12)	(0, 3)	0.51 (16)	(−3, 4)

*GHS* global health status, *Observed changes* Post-test – Pre-test; adjusted changes: Post-test – Then-test, *SD* standard deviation, *CI* confidence interval

**Table 5:** Observed and adjusted changes of the QLQ–BR23 questionnaire after three and six months

	After 3 months					After 6 months				
	Between T0 and T1					Between T1 and T2				
QLQ-BR23		Observed changes		Adjusted changes			Observed changes		Adjusted changes	
		(post-test - pre-test)		(post-test - then-test)			(post-test - pre-test)		(post-test - then-test)	
	N	Mean (SD)	95% CI	Mean (SD)	95% CI	N	Mean (SD)	95% CI	Mean (SD)	95% CI
Body image	232					232				
Little worse	70	−20.44 (27.41)	(−26, −15)	−15.40 (25.02)	(−20, −10)	47	0.41 (16.58)	(−4, 4)	−10.76 (22.82)	(−16, −5)
No change	41	−7.32 (15.16)	(−11, −3)	−4.07 (15.60)	(−8, 0)	46	2.72 (10.1)	(0, 5)	−0.91 (12.82)	(−4, 2)
Little better	49	−14.91 (24.41)	(−21, −9)	−4.93 (18.31)	(−9, −1)	65	1.28 (22.93)	(−3, 6)	−1.45 (20.88)	(−6, 3)
Sexual functioning	198					198				
Little worse	56	6.85 (20.79)	(2, 11)	7.44 (21.06)	(3, 12)	56	−0.44 (12.55)	(−4, 3)	10.53 (17.93)	(6, 15)
No change	34	3.92 (22.87)	(−3, 11)	5.88 (22.80)	(−1, 13)	34	2.03 (16.33)	(−2, 6)	2.44 (16.48)	(−2, 7)
Little better	43	1.94 (15.94)	(−2, 6)	0 (10.91)	(−3, 3)	43	−0.89 (18.1)	(−5, 3)	0 (17.70)	(−4, 4)
Sexual enjoyment	74					74				
Little worse	23	14.49 (19.66)	(7, 22)	11.59 (19.09)	(5, 18)	23	12.50 (29.5)	(0, 25)	14.58 (20.97)	(5, 24)
No change	16	10.42 (33.82)	(−4, 25)	4.17 (29.50)	(−9, 17)	16	1.45 (21.27)	(−6, 9)	2.90 (28.27)	(−7, 13)
Little better	11	12.12 (22.47)	(0, 24)	9.09 (21.56)	(−3, 21)	11	−2.08 (22.67)	(−12, 8)	0 (21.08)	(−9, 9)
Future perspectives	236					236				
Little worse	68	7.35 (32.5)	(1, 14)	−8.82 (30.81)	(−15, −3)	68	3.47 (30.16)	(−4, 11)	−0.69 (24.30)	(−7, 5)
No change	43	20.93 (32.55)	(13, 29)	7.75 (26.06)	(1, 14)	43	0 (23.57)	(−6, 6)	4.76 (30.43)	(−3, 12)
Little better	48	10.42 (30.1)	(3, 18)	3.47 (24.06)	(−2, 9)	48	1.06 (25.38)	(−4, 6)	8.47 (23.92)	(3, 13)
Systemic therapy side effects	252					252				
Little worse	72	18.83 (21.51)	(15, 23)	19.79 (18.60)	(16, 23)	72	−3.49 (18.26)	(−8, 1)	10.27 (18.90)	(6, 15)
No change	45	4.41 (15.02)	(1, 8)	3.84 (14.03)	(0, 7)	45	0.23 (10.2)	(−2, 3)	2.45 (7.89)	(1, 4)
Little better	54	6.02 (13.64)	(3, 9)	6.02 (12.35)	(3, 9)	54	−4.19 (15.12)	(−7, −1)	3.94 (19.39)	(0, 8)
Breast symptoms	212					212				
Little worse	57	14.08 (27.69)	(8, 2)	18.08 (22.21)	(13, 23)	57	−4.31 (16.81)	(−8, 0)	6.44 (18.26)	(2, 11)
No change	36	10.34 (20.43)	(5, 16)	9.72 (21.59)	(4, 16)	36	−3.57 (13.35)	(−7, 0)	3.77 (14.28)	(0, 7)
Little better	53	16.61 (21.1)	(12, 21)	7.13 (18.87)	(3, 11)	53	−2.21 (20.17)	(−7, 2)	3.63 (20.65)	(−1, 8)
Arm symptoms	235					235				
Little worse	67	7.88 (17.6)	(4, 11)	8.54 (19.28)	(5, 12)	67	0.58 (18.27)	(−4, 5)	10.76 (19.53)	(6, 15)
No change	41	3.39 (13.54)	(0, 7)	4.74 (14.14)	(1, 8)	41	1.89 (13.82)	(−1, 5)	5.89 (14.65)	(2, 9)
Little better	55	4.55 (17.51)	(1, 8)	1.41 (13.83)	(−2, 5)	55	0.69 (13.98)	(−2, 4)	0.69 (16.93)	(−3, 4)

*Observed changes* Post-test – Pre-test; adjusted changes: Post-test – Then-test

*SD* standard deviation, *CI* confidence interval

Based on the scales that have at the same time an increase of the RS effect in case of deterioration and a decrease of the RS effect in case of improvement (GHS, role, cognitive, social and sexual functioning, pain, insomnia, diarrhea, body image, breast and arm symptoms), the minimal and maximal MID for observed and adjusted MID were calculated in case of deterioration and improvement of HRQOL after three and six months (Table 6). The diarrhea scale was excluded from the analysis because it will disrupt the results; due to a number of scores containing zero values. The financial difficulties and appetite loss dimensions were also added since a remarkable increase was observed when deteriorating and a relatively low increase was highlighted when improving.

**Table 6:** MID in case of deterioration and improvement of HRQOL for both QLQ-C30 and QLQ-BR23 questionnaires after three and six months

	Deterioration of HRQOL		Improvement of HRQOL	
	Observed changes	Adjusted changes	Observed changes	Adjusted changes
QLQ-BR23 QLQ - C30				
After 3 months	5–26	8–19	0.3–10	0.6–4
After 6 months	0.5–10	4–18	0.8–7	0–7
After 3 months	7–20	7–18	2–15	0–7
After 6 months	0.4–4	6–11	0.7–2	0–4

A comparison of the observed and adjusted changes in case of a small deterioration (category “little worse”) to a threshold of 5 points for both the QLQ-C30 and QLQ-BR23 questionnaires after three and six months was presented in Table 7.

**Table 7:** MID for QLQ-C30 and QLQ-BR23 questionnaires after 3 and 6 months compared to a threshold of 5 points

	After 3 months		After 6 months	
	Observed changes	Adjusted changes	Observed changes	Adjusted changes
QLQ-C30				
Global Health Status	16	12^a^	0.5	8^a^
Physical functioning	12	12^a^	0.4	9^a^
Role functioning	26	19^a^	2	18^a^
Cognitive functioning	5	11^a^	1	10^a^
Social functioning	20	15^a^	1	10^a^
Emotional functioning	3.5	6^a^	0.5	6^a^
Fatigue	27	23^a^	0.2	20^a^
Pain	14	9^a^	2	17^a^
Dyspnea	9	11^a^	4	8^a^
Insomnia	6	8^a^	4	15^a^
Financial difficulties	10	8^a^	3	5^a^
Appetite loss	10	11^a^	10	4
Constipation	15	10^a^	1	3
Nausea and vomiting	9	8^a^	3	1
QLQ-BR23				
Body image	20	15^a^	0.4	11^a^
Sexual functioning	7	7^a^	0.4	11^a^
Systemic therapy side effects	19	20^a^	3	10^a^
Breast symptoms	14	18^a^	4	6^a^
Arm symptoms	8	9^a^	0.6	11^a^
Sexual enjoyment	14	12^a^	13	15^a^
Future perspectives	7	9^a^	3	0.7

^a^ indicates that the MID stayed or became greater than 5 points when the response shift effect was taken into account

**Fig. 2:**
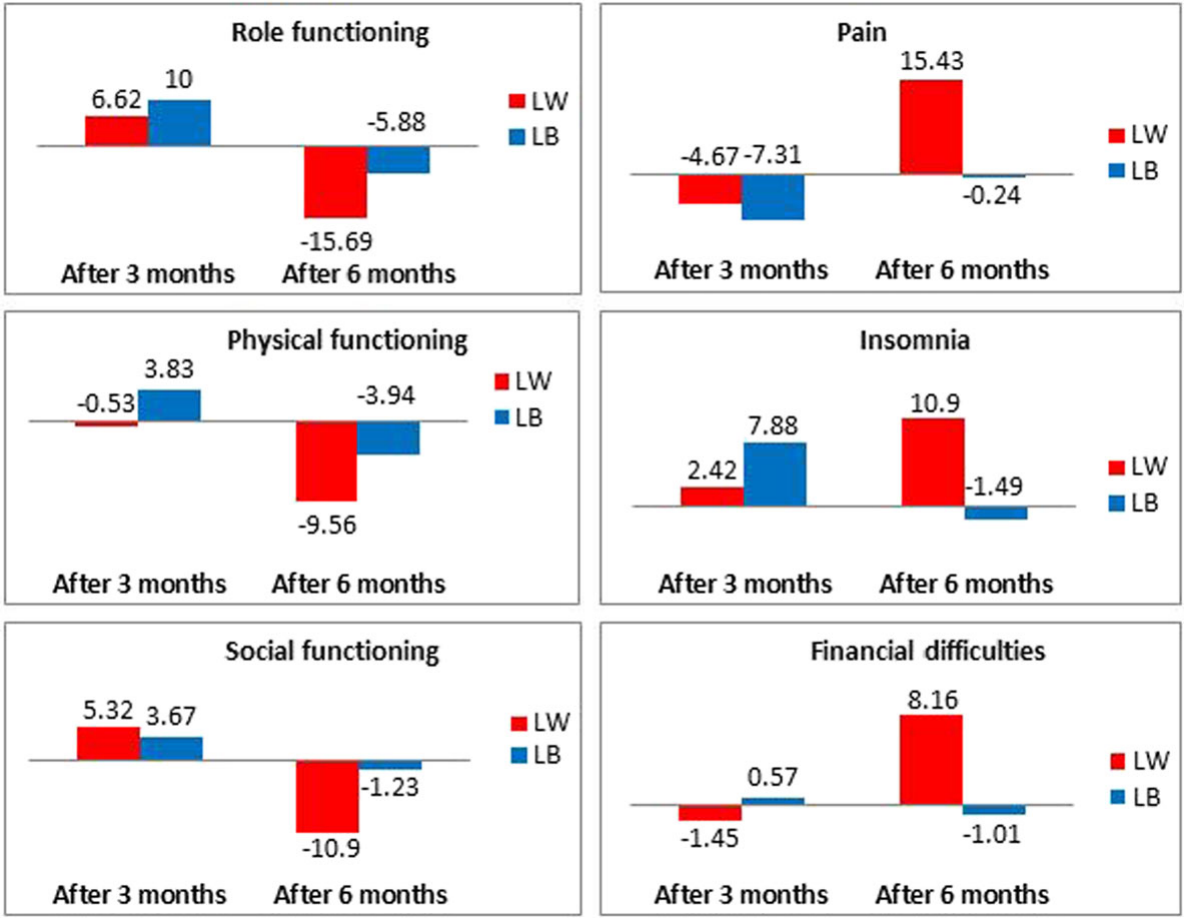
Bar charts representing the change of RS effect in the both cases: deterioration (little worse) and improvement (little better) for some dimensions of QLQ-C30 questionnaire between three and six months

**Fig. 3:**
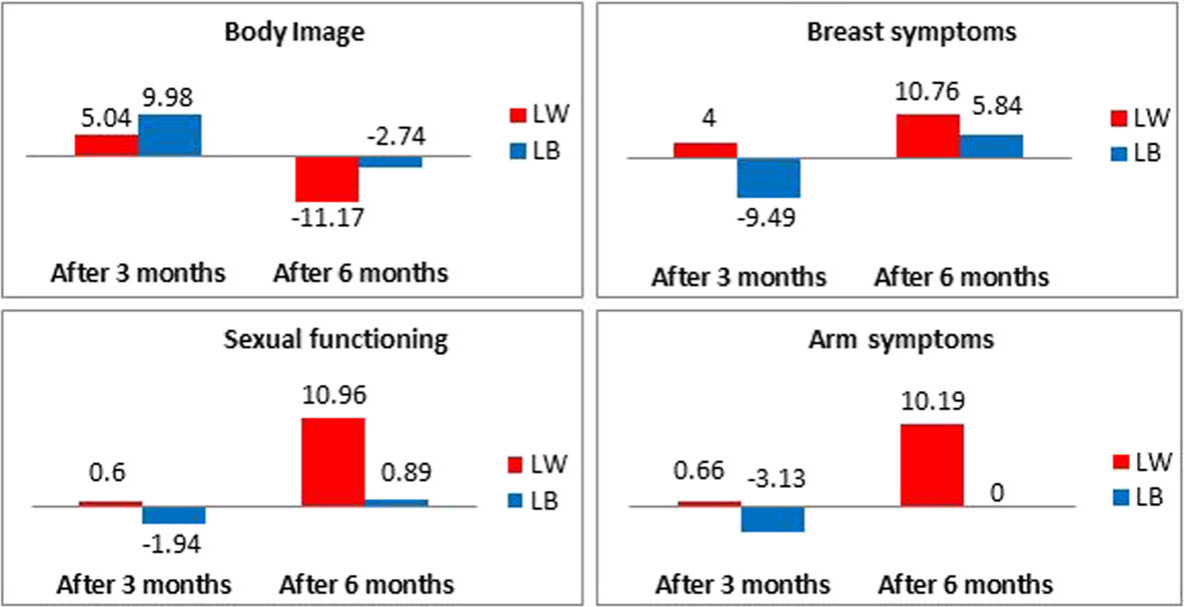
Bar charts representing the change of RS effect in the both cases: deterioration (little worse) and improvement (little better) for some dimensions of QLQ-BR23 questionnaire between three and six months

The variations of the RS effect between 3 and 6 months were represented on Figs. 2 and 3 for several HRQOL dimensions. For example, the Fig. 2 illustrates an increase of the RS effect in mean in case of a small deterioration and a decrease in case of a small improvement for the most dimensions of the QLQ-C30. Some consistent results for the most dimensions of the QLQ-BR23 questionnaire were also detected and illustrated in Fig. 3.

## Discussion

The objective of this study was to explore the impact of the occurrence of the response shift effect on the determination of the MID over time in breast cancer patients using both the QLQ-C30 and QLQ-BR23 questionnaires.

Both an increase of the RS effect in case of deterioration and a decrease of the RS effect in case of improvement was observed for 7/15 dimensions of the QLQ-C30 questionnaire (global health status (GHS), role, cognitive and social functioning, pain, insomnia, diarrhea) and 4/7 dimensions for the QLQ-BR23 questionnaire (body image, sexual function, breast and arm symptoms). This indicates the differential occurrence of the RS effect according to the change in patient’s HRQOL level over time (i.e. deterioration or improvement of HRQOL).

For 13/15 dimensions of the QLQ-C30 questionnaire (except the emotional functioning and the constipation dimensions) and 5/7 dimensions of the QLQ-BR23 questionnaire (except the sexual enjoyment and the future perspectives dimensions), an increase of the RS effect was observed in case of deterioration between T1 and T2. The RS effect becomes increasingly important over time specifically for the anchor category « little worse ». This indicates that the RS must be considered when determining the MID for deterioration.

For 8/15 dimensions of QLQ-C30 questionnaire (GHS, role, emotional, social and cognitive functioning, pain, diarrhea and insomnia dimensions) and 5/7 dimensions of the QLQ-BR23 questionnaire (except the sexual enjoyment and the future perspectives dimensions), a decrease of the RS effect was observed in case of improvement with corresponding ES values close to zero and *p*-values ≥ 0.05. These values mean that the impact of the RS effect became negligible on the determination of the MID in case of improvement after six months.

Regarding the QLQ-C30 questionnaire, the observed MID in case of deterioration after three months was between 5 and 26 points: a mean difference of 5 points was sufficient to conclude that the difference was clinically significant at T1. After taking into account the RS effect, the minimal difference considered as important for patients increased to 8 points. Furthermore, the adjusted MID became between 8 and 19 points and thus more restricted than it was for the observed changes (without taking into account the RS effect). However, at T2, the observed MID was between 0.5 and 10 then became between 4 and 18 for the adjusted MID. Thus, if we did not have taken into account the occurrence of the RS effect, we can wrongly conclude that a deterioration of 0.5 point is considered as the MID for the patients which seems to be very low. Whereas, after taking into account the RS effect, the mean difference became between 4 and 18 points which seems to be more relevant than the previous interval.

Comparing the results of adjusted MID in case of deterioration at T1 and T2, we find that the MID was between 8 and 19 points at T1 then became between 4 and 18 points at T2, which means that a smaller change of HRQOL can be considered as clinically significant to the patients. In other words, a difference of 4 points out of 100 was not enough to say that this difference was significant after three months; but the same difference became significant to the patient after 6 months. In addition, consistent results have been found for the QLQ-BR23 questionnaire in case of deterioration concerning the observed and the adjusted MID.

Regarding HRQOL improvement, no impact of the RS on the determination of the MID was observed. In contrast, the RS effect seemed to highly impact the MID for deterioration. To illustrate, the minimal of observed MID was smaller than one point in case of deterioration for QLQ-C30 (MID: 0.5–10) and QLQ-BR23 (MID: 0.4–4) after six months, the minimal of each MID was equal to 4 points for the QLQ-C30 and to 6 points for QLQ-BR23 after taking into account the RS effect. Thus, without taking into account the occurrence of the RS effect, we can wrongly conclude that a difference of less than 1 point is clinically significant to the patients.

For patients who have an improvement in their HRQOL, the minimal of observed and adjusted MID found for the two questionnaires after three and six months is close to zero and they all stayed close to zero after taking into account the RS effect, except for the observed MID after three months for the QLQ-BR23 questionnaire which was equal to 2 points. We can conclude that a very small improvement over time can be considered as important for the patient.

For thirteen over the 15 scales of the QLQ-C30 questionnaire and all scales of the QLQ-BR23, the observed and adjusted MID was greater than 5 points after three months. Whereas, after six months, 11/15 scales of the QLQ-C30 and 5/7 scales of the QLQ-BR23 had an observed MID smaller than 5 points and became greater than 5 points after taking into account the RS effect. Thus, the RS effect seems to have an important impact on the determination of the MID and notably after six months.

Our study confirms the earlier results released by Kvam and al. between two measurement times which showed that the RS has an important impact on the results in case of deterioration and was unimportant in case of improvement [13]. However, this previous study was limited to two measurement times.

Three measurement times were considered in our study allowing us to evaluate the change of RS effect and to detect specifically the dimensions for which an increase or a decrease of the RS effect was observed over time. Moreover, using three measurement times allowed us to compare the MID at three and six months and to conclude the importance of taking into account the RS effect in order to obtain a reduced interval of MID and a MID significant after six months comparing with a threshold of five points.

Another strength of our study was the consideration of all dimensions of both QLQ-C30 and QLQ-BR23 breast cancer module. The study of Kvam and al. was on patients with multiple myeloma and the QLQ-MY20 multiple myeloma module [22] was not yet validated at the time of the conception of this study, which justifies the limitation to the QLQ-C30.

Twenty two dimensions have been analysed in our study that provides to collect a lot of information on dimension impacted by the RS effect and trends over time.

A limitation of our study is the use of the then-test method to assess the occurrence of the RS effect. This method required to be planned at the time of the conception of the study and may be subject to a recall bias. Moreover, it focused on the recalibration component to study the impact of the RS, thus we recommend more researches in order to determine the impact of the other components of the RS effect (reprioritization and reconceptualization) on the determination of the MID. The structural equation modeling may be preferable to the then-test method to detect all the three components of the RS in a HRQOL analysis. However, the Oort procedure based on the structural equation modelling to detect the RS effect was developed and mainly applied the SF-36 questionnaire [11, 23]. Some researches are still ongoing to adapt this procedure to the EORTC questionnaires [24].

Using item response theory (IRT) may be very important for the future researches to assess the components of RS and its impact on the determination of the MID [19].

Although the use of three measurement times was useful in this study, but there is a recall bias may affect the answers of patients over time. In addition, using just the anchor based approach to compute MID may bias the finding as the standard practice is the combination with both anchor and distribution based methods. The limited number of patients per anchor category is considered also as a limitation of our study. Hence, further studies are needed to study the RS trends with a quite large number of patients per anchor-item category.

The heterogeneity of our data is considered also as a limitation of this study.

The MID allows planning studies in order to determine both the required sample size and the statistical power. The MID also has a direct impact on the time to HRQOL score deterioration approach for longitudinal analysis [25, 26].

## Conclusion

Our work has extended the research for the first time to investigate the RS effect on the determination of the MID between three measurement times. This investigation permitted to detect the important role of the RS effect on the determination and the significance of the MID in the most scales of two questionnaires six months after the breast cancer diagnosis. Finally, we recommend further researches to confirm, support or review our findings and develop novel methods in order to progress in this field.

## Additional file

Additional file 1: Table S1. The Response shift effect for the QLQ-C30 questionnaire after three and six months for all patients and according to each category of the anchor. **Table S2.** The Response shift effect for the QLQ-BR23 questionnaire after three and six months for all patients and according to each category of the anchor. **Table S3.** Observed and adjusted changes of the QLQ–C30 questionnaire after three and six months. **Table S4.** Observed and adjusted changes of the QLQ–BR23 questionnaire after three and six months. (DOCX 95 kb)

## Abbreviations

EORTC: European organization for research and treatment of cancer
ES: Effect size
GHS: Global health status
HRQOL: Health-related quality of life
MID: Minimal important difference
RS: Response shift
SD: Standard deviation
