# Supplementary material for: Impact of the occurrence of a response shift on the determination of the minimal important difference in a health-related quality of life score over time
Source: Health Qual Life Outcomes. 2016 Dec 3;14:167. doi: 10.1186/s12955-016-0569-5 (PMC5135836; doi:10.1186/s12955-016-0569-5)
Supplement: Additional file 1: Table S1. — The Response shift effect for the QLQ-C30 questionnaire after three and six months for all patients and according to each category of the anchor. Table S2. The Response shift effect for the QLQ-BR23 questionnaire after three and six months for all patients and according to each category of the anchor. Table S3. Observed and adjusted changes of the QLQ–C30 questionnaire after three and six months. Table S4. Observed and adjusted changes of the QLQ–BR23 questionnaire after three and six months. (DOCX 95 kb) [file 12955_2016_569_MOESM1_ESM.docx]

**Table S1:** The Response shift effect for the QLQ-C30 questionnaire after three and six months for all patients and according to each category of the anchor

|  | **After 3 months** | | | | | **After 6 months** | | | | |
| --- | --- | --- | --- | --- | --- | --- | --- | --- | --- | --- |
|  | **Between T0 and T1** | | | | | **Between T1 and T2** | | | | |
|  | **N** | **RS (SD)** | **95%CI** | **P-value** | **ES** | **N** | **RS (SD)** | **95% CI** | **P-value** | **ES** |
| **GHS** | 251 | 3.82 (17.85) |  | 0.02 | 0.20 | 251 | -0.03 (21.04) |  | 0.97 | 0 |
| Much worse | 30 | -1.94 (18.66) | (-8, 4) | 0.70 | -0.08 | 22 | -14.39 (17.29) | (-21, -8) | 0.02 | -0.90 |
| Little worse | 71 | 3.87 (15.86) | (1, 7) | 0.24 | 0.25 | 51 | -8.33 (19.22) | (-13, -4) | 0.03 | -0.42 |
| No change | 43 | 6.78 (15.67) | (3, 11) | 0.08 | 0.40 | 50 | 8 (19.04) | (3, 13) | 0.03 | 0.42 |
| Little better | 58 | 1.29 (17.51) | (-3, 5) | 0.67 | 0.08 | 67 | -0.25 (21.61) | (-5, 4) | 0.80 | -0.01 |
| Much better | 49 | 7.65 (22.56) | (2, 13) | 0.08 | 0.39 | 61 | 5.74 (24.93) | (0, 11) | 0.12 | 0.30 |
| **Physical functioning** | 255 | 1.54 (12.95) |  | 0.30 | 0.11 | 255 | -5.2 (12.86) |  | 0 | -0.32 |
| Much worse | 30 | -1.33 (10.56) | (-5, 2) | 0.98 | -0.06 | 22 | -16.97 (18.83) | (-24, -10) | 0 | -1.10 |
| Little worse | 73 | -0.53 (11.11) | (-3, 2) | 0.49 | -0.05 | 53 | -9.56 (10.42) | (-12, -7) | 0 | -0.52 |
| No change | 46 | 1.30 (13.67) | (-2, 5) | 0.17 | 0.09 | 51 | -2.29 (8.5) | (-4, 0) | 0.13 | -0.26 |
| Little better | 57 | 3.83 (14.47) | (1, 7) | 0.45 | 0.28 | 69 | -3.94 (14.71) | (-7, -1) | 0.04 | -0.25 |
| Much better | 49 | 3.95 (14.71) | (0, 7) | 0.16 | 0.35 | 60 | -0.94 (14.42) | (-4, 2) | 0.72 | -0.07 |
| **Role functioning** | 253 | 6.06 (21.71) |  | 0 | 0.32 | 253 | -8.17 (27.07) |  | 0 | -0.29 |
| Much worse | 30 | 1.67 (28.48) | (-7, 11) | 0.50 | 0.06 | 23 | -27.54 (36.45) | (-41, -14) | 0 | -1.05 |
| Little worse | 73 | 6.62 (19.98) | (3, 11) | 0.04 | 0.46 | 51 | -15.69 (28.95) | (-22, -9) | 0 | -0.52 |
| No change | 46 | 3.26 (16.34) | (-1, 7) | 0.07 | 0.19 | 51 | -1.96 (18.45) | (-6, 2) | 0.67 | -0.11 |
| Little better | 55 | 10 (26.57) | (4, 16) | 0.05 | 0.45 | 68 | -5.88 (26.67) | (-11, 0) | 0.24 | -0.21 |
| Much better | 49 | 6.12 (19.75) | (1, 11) | 0.16 | 0.39 | 60 | -2.22 (29.67) | (-9, 4) | 0.98 | -0.09 |
| **Emotional functioning** | 254 | -7.56 (21.03) |  | 0 | -0.29 | 254 | 2.20 (22.68) |  | 0.20 | 0.09 |
| Much worse | 30 | -14.26 (21.73) | (-21, -8) | 0.07 | -0.48 | 22 | -13.89 (27.74) | (-24, -4) | 0.05 | -0.57 |
| Little worse | 72 | -9.34 (20.97) | (-13, -5) | 0 | -0.38 | 52 | -5.66 (19.77) | (-10, -1) | 0.16 | -0.24 |
| No change | 44 | -8.52 (17.14) | (-13, -4) | 0.03 | -0.42 | 50 | 8.94 (21.58) | (4, 14) | 0.04 | 0.47 |
| Little better | 59 | -11.11 (23.15) | (-16, -6) | 0.01 | -0.43 | 69 | 5.68 (21.83) | (1, 10) | 0.10 | 0.23 |
| Much better | 49 | 4.31 (21.64) | (-1, 9) | 0.22 | 0.17 | 61 | 5.24 (25.22) | (0, 11) | 0.23 | 0.21 |
| **Cognitive functioning** | 254 | -3.94 (16.85) |  | 0.01 | -0.19 | 254 | -3.08 (20.03) |  | 0.04 | -0.15 |
| Much worse | 30 | -7.22 (15.59) | (-12, -2) | 0.22 | -0.29 | 22 | -9.09 (30.31) | (-20, 2) | 0.08 | -0.42 |
| Little worse | 73 | -5.48 (20.04) | (-9, -2) | 0.08 | -0.26 | 52 | -10.58 (21.9) | (-16, -5) | 0.02 | -0.42 |
| No change | 44 | -1.52 (10.67) | (-4, 1) | 0.69 | -0.11 | 50 | -2.33 (16.5) | (-6, 2) | 0.35 | -0.16 |
| Little better | 57 | -5.85 (19.29) | (-10, -2) | 0.08 | -0.27 | 69 | 0.97 (18.28) | (-3, 5) | 0.98 | 0.05 |
| Much better | 50 | 0.33 (15.61) | (-3, 4) | 0.86 | 0.02 | 61 | 0.27 (19.6) | (-4, 4) | 0.98 | 0.01 |
| **Social functioning** | 249 | 5.09 (19.43) |  | 0 | 0.28 | 249 | -5.96 (24.47) |  | 0.01 | -0.23 |
| Much worse | 26 | 8.33 (22.73) | (1, 16) | 0.28 | 0.34 | 21 | -16.67 (39.44) | (-32, -2) | 0.06 | -0.54 |
| Little worse | 72 | 5.32 (19.94) | (1, 9) | 0.05 | 0.39 | 52 | -10.90 (25.54) | (-17, -5) | 0.02 | -0.40 |
| No change | 42 | 3.17 (14.37) | (-1, 7) | 0.08 | 0.24 | 49 | -4.76 (16.67) | (-9, -1) | 0.40 | -0.22 |
| Little better | 59 | 3.67 (20.55) | (-1, 8) | 0.59 | 0.18 | 68 | -1.23 (24.32) | (-6, 4) | 0.91 | -0.05 |
| Much better | 50 | 6.33 (19.89) | (2, 11) | 0.06 | 0.33 | 59 | -4.24 (24.87) | (-10, 1) | 0.24 | -0.20 |
| **Financial difficulties** | 240 | -1.11 (15.32) |  | 0.30 | -0.08 | 240 | 2.50 (16.26) |  | 0.22 | 0.12 |
| Much worse | 26 | -2.56 (29.70) | (-13, 7) | 0.33 | -0.13 | 21 | 12.70 (30.69) | (1, 24) | 0.17 | 0.39 |
| Little worse | 69 | -1.45 (12.04) | (-4, 1) | 0.58 | -0.14 | 49 | 8.16 (24.09) | (2, 14) | 0.13 | 0.30 |
| No change | 41 | -1.63 (7.27) | (-4, 0) | 0.31 | -0.31 | 47 | -0.71 (4.86) | (-2, 0) | 0.57 | -0.15 |
| Little better | 58 | 0.57 (15.91) | (-3, 4) | 0.86 | 0.03 | 66 | -1.01 (16.51) | (-4, 2) | 0.82 | -0.05 |
| Much better | 46 | -1.45 (18.53) | (-6, 3) | 0.98 | -0.12 | 57 | 0.58 (13.35) | (-2, 4) | 0.60 | 0.05 |
| **Fatigue** | 254 | -1.20 (19.42) |  | 0.51 | -0.06 | 254 | 10.83 (23.93) |  | 0 | 0.41 |
| Much worse | 29 | 9.20 (16.82) | (4, 15) | 0.19 | 0.32 | 23 | 27.29 (29.15) | (17, 38) | 0.01 | 0.98 |
| Little worse | 73 | -4.57 (21.36) | (-9, 0) | 0.31 | -0.33 | 53 | 19.92 (23.23) | (15, 25) | 0 | 0.85 |
| No change | 46 | -2.17 (15.29) | (-6, 2) | 0.62 | -0.12 | 51 | 3.70 (20.08) | (-1, 8) | 0.32 | 0.20 |
| Little better | 57 | -4.58 (21.29) | (-9, 0) | 0.35 | -0.23 | 68 | 9.97 (23.76) | (5, 15) | 0.01 | 0.44 |
| Much better | 49 | 2.49 (19.77) | (-2, 7) | 0.71 | 0.12 | 59 | 3.39 (26.04) | (-2, 9) | 0.61 | 0.13 |
| **Nausea and vomiting** | 255 | -1.37 (13.59) |  | 0.26 | -0.13 | 255 | 3.59 (17.65) |  | 0 | 0.19 |
| Much worse | 29 | -0.57 (10.43) | (-4, 3) | 0.78 | -0.10 | 23 | 10.87 (27.8) | (1, 21) | 0.04 | 0.40 |
| Little worse | 74 | -0.45 (15.35) | (-3, 3) | 0.71 | -0.05 | 53 | 4.09 (14.58) | (1, 7) | 0.09 | 0.26 |
| No change | 46 | 0.72 (9.91) | (-2, 3) | 1 | 0.04 | 51 | 0.98 (10.23) | (-1, 3) | 0.10 | 0.10 |
| Little better | 57 | -5.26 (15.16) | (-9, -2) | 0.05 | -0.87 | 70 | 6.67 (25.44) | (2, 12) | 0.05 | 29 |
| Much better | 49 | -0.68 (14.42) | (-4, 3) | 0.38 | -0.05 | 58 | -1.15 (13.56) | (-4, 2) | 0.49 | -0.09 |
| **Pain** | 260 | -3.33 (22.51) |  | 0.14 | -0.16 | 260 | 5.83 (22.2) |  | 0 | 0.23 |
| Much worse | 32 | 0.52 (36.29) | (-10, -11) | 0.68 | 0.02 | 23 | 12.32 (25.73) | (3, 22) | 0.11 | 0.43 |
| Little worse | 75 | -4.67 (21.32) | (-9, -1) | 0.29 | -0.24 | 54 | 15.43 (26.27) | (9, 21) | 0 | 0.56 |
| No change | 46 | 1.45 (18.2) | (-3, 6) | 0.83 | 0.09 | 52 | 2.24 (13.21) | (-1, 5) | 0.30 | 0.18 |
| Little better | 57 | -7.31 (23.57) | (-13, -2) | 0.16 | -0.35 | 70 | -0.24 (23.14) | (-5, 4) | 0.80 | -0.01 |
| Much better | 50 | -3.67 (18.22) | (-8, 1) | 0.61 | -0.27 | 61 | 4.92 (23.83) | (0, 1) | 0.17 | 0.21 |
| **Dyspnea** | 251 | 1.20 (15.16) |  | 0.59 | 0.06 | 251 | 3.98 (22.4) |  | 0.03 | 0.17 |
| Much worse | 30 | 4.44 (11.52) | (-1, 8) | 0.38 | 0.2 | 23 | 18.84 (33.07) | (7, 31) | 0.04 | 0.56 |
| Little worse | 73 | 2.28 (15.04) | (-1, 5) | 0.38 | 0.11 | 52 | 3.85 (27.74) | (-3, 10) | 0.14 | 0.17 |
| No change | 45 | 0 (12.31) | (-3, 3) | 0.77 | 0 | 51 | 0.65 (10.52) | (-2, 3) | 0.79 | 0.05 |
| Little better | 55 | 0.61 (17.56) | (-3, 5) | 0.77 | 0.03 | 67 | 3.98 (22.11) | (-1, 8) | 0.51 | 0.17 |
| Much better | 48 | -0.69 (17.52) | (-5, 4) | 0.42 | -0.03 | 58 | 1.15 (24.15) | (-4, 6) | 0.60 | 0.05 |
| **Insomnia** | 249 | 6.83 (30.64) |  | 0.01 | 0.22 | 249 | 3.08 (30.41) |  | 0.30 | 0.10 |
| Much worse | 30 | 13.33 (32.28) | (3, 23) | 0.13 | 0.39 | 23 | 14.49 (37.37) | (1, 28) | 0.13 | 0.48 |
| Little worse | 69 | 2.42 (29.33) | (-3, 8) | 0.49 | 0.08 | 52 | 10.9 (37.18) | (2, 2) | 0.07 | 0.33 |
| No change | 46 | 7.25 (26.21) | (1, 14) | 0.27 | 0.25 | 50 | 3.33 (25.42) | (-3, 9) | 0.24 | 0.18 |
| Little better | 55 | 7.88 (37.93) | (-1, 16) | 0.11 | 0.27 | 67 | -1.49 (29.83) | (-8, 5) | 0.74 | -0.05 |
| Much better | 49 | 7.48 (27.44) | (1, 14) | 0.29 | 0.23 | 57 | -3.51 (26.49) | (-9, 2) | 0.36 | -0.11 |
| **Appetite loss** | 249 | 2.28 (21.05) |  | 0.23 | 0.11 | 249 | 4.69 (22.2) |  | 0.03 | 0.19 |
| Much worse | 30 | 4.44 (28.68) | (-4, 13) | 0.71 | 0.17 | 23 | 18.84 (38.7) | (5, 33) | 0.03 | 0.52 |
| Little worse | 73 | 0.91 (20.77) | (-3, 5) | 0.55 | 0.06 | 53 | 13.84 (24.84) | (8, 20) | 0 | 0.51 |
| No change | 46 | 4.35 (13.35) | (1, 8) | 0.19 | 0.22 | 50 | 0.67 (15.78) | (-3, 4) | 0.98 | 0.03 |
| Little better | 54 | 1.85 (22.82) | (-3, 7) | 0.79 | 0.09 | 68 | 1.96 (21.46) | (-2, 6) | 0.74 | 0.09 |
| Much better | 46 | 1.45 (22.17) | (-4, 7) | 0.79 | 0.06 | 55 | -3.03 (19.53) | (-7, 1) | 0.47 | -0.17 |
| **Constipation** | 247 | 0 (22.61) |  | 0.87 | 0 | 247 | 4.18 (25.89) |  | 0.05 | 0.16 |
| Much worse | 29 | 8.05 (27.68) | (-1, 17) | 0.25 | 0.27 | 21 | 11.11 (35.49) | (-2, 24) | 0.16 | 0.35 |
| Little worse | 68 | -4.90 (18.45) | (-9, -1) | 0.19 | -0.26 | 48 | 4.17 (25.38) | (-2, 10) | 0.29 | 0.16 |
| No change | 42 | 2.38 (18.61) | (-2, 7) | 0.42 | 0.11 | 48 | 2.08 (14.43) | (-1, 6) | 0.43 | 0.15 |
| Little better | 58 | 0 (27.22) | (-6, 6) | 0.87 | 0 | 70 | 2.86 (35.32) | (-4, 10) | 0.71 | 0.10 |
| Much better | 50 | 0 (23.33) | (-6, 6) | 0.71 | 0 | 60 | 5 (21.10) | (0, 1) | 0.26 | 0.18 |
| **Diarrhea** | 248 | 2.42 (14.99) |  | 0.09 | 0.15 | 248 | 0.94 (20.28) |  | 0.42 | 0.06 |
| Much worse | 29 | -1.15 (10.85) | (-5, 2) | 0.70 | -0.11 | 22 | 4.55 (18.67) | (-2, 11) | 0.90 | 0.17 |
| Little worse | 71 | 3.29 (16.09) | (0, 6) | 0.28 | 0.18 | 48 | 3.47 (14.16) | (0, 7) | 0.22 | 0.23 |
| No change | 43 | 1.55 (12.50) | (-2, 5) | 0.54 | 0.13 | 48 | -0.69 (14.57) | (-4, 3) | 0.98 | -0.07 |
| Little better | 56 | 2.98 (17.15) | (-1, 7) | 0.34 | 0.16 | 69 | 0 (26.81) | (-5, 5) | 0.86 | 0 |
| Much better | 49 | 3.40 (15.58) | (0, 7) | 0.27 | 0.22 | 61 | 0 (22.77) | (-5, 5) | 0.82 | 0 |

GHS: Global health status; RS: Response Shift; SD: Standard Deviation; CI: Confidence Interval; ES: Effect Size

**Table S2:** The Response shift effect for the QLQ-BR23 questionnaire after three and six months for all patients and according to each category of the anchor

|  | **After 3 months** | | | | | **After 6 months** | | | | |
| --- | --- | --- | --- | --- | --- | --- | --- | --- | --- | --- |
|  | **Between T0 and T1** | | | | | **Between T1 and T2** | | | | |
| QLQ-BR23 | **N** | **RS (SD)** | **95%CI** | **P-value** | ES | **N** | **RS (SD)** | **95%CI** | **P-value** | **ES** |
| **Body image** | 232 | 6.50 (20.73) |  | 0.01 | 0.37 | 232 | -7.18 (22.33) |  | 0.01 | -0.23 |
| Much worse | 28 | 6.45 (24.67) | (-1, 14) | 0.76 | 0.34 | 21 | -35.32 (38.5) | (-50, -21) | 0 | -1.11 |
| Little worse | 70 | 5.04 (17.89) | (1, 9) | 0.11 | 0.32 | 47 | -11.17 (21.1) | (-16, -6) | 0.03 | -0.37 |
| No change | 41 | 3.25 (14.54) | (-1, 7) | 0.11 | 0.20 | 46 | -3.62 (12.25) | (-7, -1) | 0.28 | -0.19 |
| Little better | 49 | 9.98 (23) | (4, 15) | 0.11 | 0.62 | 65 | -2.74 (24.7) | (-8, 2) | 0.63 | -0.09 |
| Much better | 44 | 8.02 (25.98) | (1, 15) | 0.20 | 0.38 | 53 | -1.05 (22.85) | (-6, 4) | 0.90 | -0.04 |
| **Sexual functioning** | 198 | 0.76 (15.06) |  | 0.77 | 0.03 | 198 | 4.29 (18.88) |  | 0.10 | 0.19 |
| Much worse | 26 | 5.77 (16.29) | (0, 11) | 0.40 | 0.23 | 19 | 16.67 (24.85) | (7, 27) | 0.10 | 0.95 |
| Little worse | 56 | 0.60 (15.56) | (-3, 4) | 0.90 | 0.02 | 38 | 10.96 (19.86) | (6, 16) | 0.07 | 0.52 |
| No change | 34 | 1.96 (15.77) | (-3, 7) | 0.73 | 0.08 | 41 | 0.41 (18.44) | (-4, 5) | 0.90 | 0.01 |
| Little better | 43 | -1.94 (12.71) | (-5, 1) | 0.77 | -0.08 | 56 | 0.89 (16.02) | (-3, 4) | 0.88 | 0.05 |
| Much better | 39 | -0.43 (15.53) | (-5, 4) | 0.89 | -0.02 | 44 | 1.14 (19.49) | (-4, 6) | 0.79 | 0.05 |
| **Sexual enjoyment** | 74 | -4.05 (20.74) |  | 0.26 | -0.16 | 74 | 3.15 (23.49) |  | 0.21 | 0.13 |
| Much worse | 8 | -4.17 (21.36) | (-18, 10) | 0.73 | -0.12 | 4 | 25 (16.67) | (5, 45) | 0.06 | 0 |
| Little worse | 23 | -2.90 (24.44) | (-12, 6) | 0.59 | -0.16 | 16 | 2.08 (30.96) | (-11, 16) | 0.55 | 0.10 |
| No change | 16 | -6.25 (13.44) | (-12, 0) | 0.51 | -0.23 | 23 | 1.45 (25.58) | (-8, 11) | 0.69 | 0.06 |
| Little better | 11 | -3.03 (23.35) | (-16, 10) | 0.80 | -0.10 | 16 | 2.08 (14.75) | (-4, 9) | 0.83 | 0.08 |
| Much better | 16 | -4.17 (20.64) | (-13, 5) | 0.64 | -0.17 | 15 | 2.22 (23.46) | (-8, 13) | 0.60 | 0.09 |
| **Future perspectives** | 236 | -7.91 (30.3) |  | 0.01 | -0.27 | 236 | 1.27 (30.77) |  | 0.64 | 0.04 |
| Much worse | 31 | -6.45 (31.53) | (-16, 3) | 0.36 | -0.23 | 22 | -18.18 (32.1) | (-30, -6) | 0.10 | -0.74 |
| Little worse | 68 | -16.18 (27.31) | (-22, -11) | 0 | -0.56 | 48 | -4.17 (31.98) | (-12, 4) | 0.49 | -0.13 |
| No change | 43 | -13.18 (29.22) | (-21, -6) | 0.04 | -0.47 | 49 | 4.76 (34.69) | (-4, 13) | 0.47 | 0.18 |
| Little better | 48 | -6.94 (32.95) | (-15, 1) | 0.22 | -0.28 | 63 | 7.41 (25.71) | (2, 13) | 0.18 | 0.23 |
| Much better | 46 | 7.25 (32.14) | (-1, 15) | 0.20 | 0.24 | 54 | 3.7 (31.5) | (-3, 11) | 0.52 | 0.11 |
| **Systemic therapy side effects** | 252 | 0.42 (12.32) |  | 0.73 | 0.03 | 252 | 8.16 (16.76) |  | 0 | 0.42 |
| Much worse | 32 | -0.40 (15.17) | (-5, 4) | 0.71 | -0.02 | 23 | 23.19 (21.5) | (15, 31) | 0 | 1.25 |
| Little worse | 72 | 0.96 (12.49) | (-1, 3) | 0.78 | 0.07 | 53 | 13.76 (19.15) | (9, 18) | 0 | 0.73 |
| No change | 45 | -0.56 (8.12) | (-3, 1) | 0.90 | -0.04 | 52 | 2.22 (8.64) | (0, 4) | 0.53 | 0.19 |
| Little better | 54 | 0 (12.64) | (-3, 3) | 0.47 | 0 | 65 | 8.13 (19.39) | (4, 12) | 0 | 0.45 |
| Much better | 49 | 1.52 (13.62) | (-2, 5) | 0.86 | 0.08 | 59 | 2.52 (17.02) | (-1, 6) | 0.22 | 0.15 |
| **Breast symptoms** | 212 | -2.59 (19.13) |  | 0.69 | -0.18 | 212 | 8.54 (20.19) |  | 0 | 0.35 |
| Much worse | 25 | 0.56 (18.37) | (-6, 7) | 0.60 | 0.05 | 15 | 10.56 (21) | (1, 2) | 0.14 | 0.42 |
| Little worse | 57 | 4 (17.94) | (0, 8) | 0.09 | 0.24 | 47 | 10.76 (19.94) | (6, 16) | 0.01 | 0.4 |
| No change | 36 | -0.62 (11.35) | (-4, 3) | 0.68 | -0.07 | 42 | 7.34 (16.22) | (3, 12) | 0.02 | 0.47 |
| Little better | 53 | -9.49 (21.49) | (-14, -5) | 0.12 | -0.76 | 59 | 5.84 (14.8) | (3, 9) | 0.17 | 0.23 |
| Much better | 41 | -6.50 (25.04) | (-13, 0) | 0.08 | -0.36 | 49 | 10.09 (30.08) | (3, 17) | 0.04 | 0.39 |
| **Arm symptoms** | 235 | -1.94 (16.33) |  | 0.50 | -0.14 | 235 | 3.81 (19.17) |  | 0.02 | 0.21 |
| Much worse | 29 | -2.49 (20.66) | (-9, 4) | 0.96 | -0.15 | 18 | 6.17 (17.14) | (-1, 13) | 0.20 | 0.29 |
| Little worse | 67 | 0.66 (18) | (-3, 4) | 0.55 | 0.05 | 48 | 10.19 (20.06) | (5, 15) | 0 | 0.47 |
| No change | 41 | 1.36 (7.93) | (-1, 3) | 0.31 | 0.16 | 50 | 4 (10.88) | (1, 7) | 0.01 | 0.36 |
| Little better | 55 | -3.13 (18.07) | (-7, 1) | 0.45 | -0.18 | 64 | 0 (17.51) | (-4, 4) | 0.71 | 0 |
| Much better | 43 | -7.24 (16.6) | (-11, -3) | 0.02 | -0.91 | 55 | 1.72 (15.71) | (-2, 5) | 0.18 | 0.13 |

RS: Response Shift; SD: Standard Deviation; CI: Confidence Interval; ES: Effect Size

**Table S3:** Observed and adjusted changes of the QLQ–C30 questionnaire after three and six months

|  | **After 3 months** | | | | | **After 6 months** | | | | |
| --- | --- | --- | --- | --- | --- | --- | --- | --- | --- | --- |
|  | **Between T0 and T1** | | | | | **Between T1 and T2** | | | | |
|  |  | **Observed changes** | | **Adjusted changes** | |  | **Observed changes** | | **Adjusted changes** | |
|  |  | **(post-test - pre-test)** | | **(post-test - then-test)** | |  | **(post-test - pre-test)** | | **(post-test - then-test)** | |
|  | **N** | **Mean (SD)** | **95% CI** | **Mean (SD)** | **95% CI** | **N** | **Mean (SD)** | **95% CI** | **Mean (SD)** | **95% CI** |
| **GHS** | 251 |  |  |  |  | 251 |  |  |  |  |
| Much worse | 30 | -28.33 (26.86) | (-37, -20) | -30.28 ( 22.48) | (-37, -23) | 22 | -7.2 (20.46) | (-15, 0) | -21.59 ( 24.62) | (-31, -13) |
| Little worse | 71 | -16.31 (16.99) | (-20, -13) | -12.44 ( 16.78) | (-16, -9) | 51 | 0.49 (15.4) | (-3, 4) | -7.84 ( 15.22) | (-11, -4) |
| No change | 43 | -2.13 (16.17) | (-6, 2) | 4.65 ( 17.85) | (0, 9) | 50 | -1 (16.12) | (-5, 3) | 7 ( 14.71) | (4, 10) |
| Little better | 58 | 0.29 (18.4) | (-4, 4) | 1.58 ( 14.93) | (-2, 5) | 67 | 7.21 (17.22) | (4, 11) | 6.97 ( 16.70) | (4, 10) |
| Much better | 49 | 7.31 (21.69) | (2, 13) | 14.97 ( 20.41) | (10, 20) | 61 | 15.16 (17.7) | (11, 19) | 20.90 ( 18.55) | (17, 25) |
| **Physical functioning** | 255 |  |  |  |  | 255 |  |  |  |  |
| Much worse | 30 | -24.67 (15.87) | (-30, -20) | -26 ( 14.26) | (-30, -22) | 22 | -6.06 (15.92) | (-12, 0) | -23.03 ( 19.05) | (-30, -16) |
| Little worse | 73 | -11.76 (10.99) | (-14, -10) | -12.28 ( 12.71) | (-15, -10) | 53 | 0.38 (11.43) | (-2, 3) | -9.18 ( 12.83) | (-12, -6) |
| No change | 46 | -3.99 (10.17) | (-7, -1) | -2.68 ( 12.76) | (-6, 0) | 51 | 0.72 (6.97) | (-1, 2) | -1.57 ( 6.34) | (-3, 0) |
| Little better | 57 | -4.53 (12.89) | (-7, -2) | -0.70 ( 11.86) | (-3, 2) | 69 | 0.85 (13.02) | (-2, 3) | -3.09 ( 14.03) | (-6, 0) |
| Much better | 49 | -1.90 (10.89) | (-5, 1) | 2.04 ( 14.29) | (-1, 5) | 60 | 4.53 (11.63) | (2, 7) | 3.58 ( 11.34) | (1, 6) |
| **Role functioning** | 253 |  |  |  |  | 253 |  |  |  |  |
| Much worse | 30 | -59.44 (29.91) | (-69, -50) | -57.78 ( 24.66) | (-65, -50) | 23 | -7.25 (28.79) | (-18, 3) | -34.78 ( 32.53) | (-46, -23) |
| Little worse | 73 | -25.57 (21.71) | (-30, -21) | -18.95 ( 23.78) | (-24, -14) | 51 | -1.96 (29.18) | (-9, 5) | -17.65 ( 29.14) | (-24, -11) |
| No change | 46 | -2.90 (19.66) | (-8, 2) | 0.36 ( 21.80) | (-5, 6) | 51 | 3.92 (15.49) | (0, 8) | 1.96 ( 19.05) | (-3, 6) |
| Little better | 55 | -9.09 (20.74) | (-14, -4) | 0.91 ( 24.72) | (-5, 6) | 68 | 5.88 (21.89) | (1, 1) | 0 ( 23.92) | (-5 ,5) |
| Much better | 49 | -2.38 (20.41) | (-7, 3) | 3.74 ( 20.49) | (-1, 9) | 60 | 14.17 (27.4) | (8, 2) | 11.94 ( 22.57) | (7, 17) |
| **Emotional functioning** | 254 |  |  |  |  | 254 |  |  |  |  |
| Much worse | 30 | -2.96 (34.81) | (-14, 8) | -17.22 ( 27.93) | (-26, -9) | 22 | -3.16 (25.48) | (-13, 6) | -17.05 ( 32.07) | (-29, -5) |
| Little worse | 72 | 3.05 (24.09) | (-2, 8) | -6.29 ( 22.02) | (-11, -2) | 52 | -0.53 (20.19) | (-5, 4) | -6.20 ( 16.43) | (-10, -2) |
| No change | 44 | 19.44 (18.31) | (15, 24) | 10.92 ( 18.26) | (6, 16) | 50 | -0.78 (15.55) | (-4, 3) | 8.17 ( 21.26) | (3, 13) |
| Little better | 59 | 15.07 (26.33) | (9, 21) | 3.95 ( 19.51) | (0, 8) | 69 | 1.53 (20.05) | (-2, 6) | 7.21 ( 19.13) | (3, 11) |
| Much better | 49 | 10.60 (22.75) | (5, 16) | 14.91 ( 23.64) | (9, 21) | 61 | 12.93 (23.7) | (8, 18) | 18.17 ( 22.18) | (13, 23) |
| **Cognitive functioning** | 254 |  |  |  |  | 254 |  |  |  |  |
| Much worse | 30 | -15.56 (23.95) | (-23, -8) | -22.78 ( 24.56) | (-30, -15) | 22 | -9.09 (28.04) | (-19, 1) | -18.18 ( 24.08) | (-27, -9) |
| Little worse | 73 | -5.48 (19.46) | (-9, -2) | -10.96 ( 18.26) | (-15, -7) | 52 | 0.96 (17.9) | (-3, 5) | -9.62 ( 19.06) | (-14, -5) |
| No change | 44 | 0.76 (13.43) | (-3, 4) | -0.76 ( 12.43) | (-4, 2) | 50 | 2.67 (12.31) | (0, 6) | 0.33 ( 15.97) | (-3, 4) |
| Little better | 57 | 5.26 (21.4) | (1, 1) | -0.58 ( 17.24) | (-4, 3) | 69 | 3.62 (13.37) | (1, 6) | 4.59 ( 17.82) | (1, 8) |
| Much better | 50 | 2 (13.74) | (-1, 5) | 2.33 ( 14.68) | (-1, 6) | 61 | 5.46 (13.17) | (3, 8) | 5.74 ( 15.78) | (2, 9) |
| **Social functioning** | 249 |  |  |  |  | 249 |  |  |  |  |
| Much worse | 26 | -48.72 (30.16) | (-59, -39) | -40.38 ( 36.26) | (-53, -28) | 21 | -15.87 (34.4) | (-29, -3) | -32.54 ( 39.96) | (-48, -18) |
| Little worse | 72 | -20.37 (21.34) | (-25, -16) | -15.05 ( 14.84) | (-18, -12) | 52 | 0.64 (24.47) | (-5, 6) | -10.26 ( 20.39) | (-15, -6) |
| No change | 42 | -3.97 (21.08) | (-9, 2) | -0.79 ( 17.25) | (-5, 4) | 49 | 4.42 (19.18) | (0, 9) | -0.34 ( 12.50) | (-3, 3) |
| Little better | 59 | -6.78 (19.6) | (-11, -3) | -3.11 ( 18.95) | (-7, 1) | 68 | 3.43 (20.68) | (-1, 8) | 2.21 ( 21.34) | (-2, 7) |
| Much better | 50 | 0.33 (23.2) | (-5, 6) | 6.67 ( 17.17) | (3, 11) | 59 | 11.9 (23.58) | (7, 17) | 7.63 ( 15.26) | (4, 11) |
| **Fatigue** | 154 |  |  |  |  | 254 |  |  |  |  |
| Much worse | 29 | 43.68 (33.72) | (33, 54) | 52.87 ( 28.74) | (44, 62) | 23 | 6.28 (38.17) | (-7, 20) | 33.57 ( 37.17) | (20, 47) |
| Little worse | 73 | 27.32 (21.87) | (23, 32) | 22.75 ( 22.38) | (18, 27) | 53 | 0.21 (16.34) | (-4, 4) | 20.13 ( 24.17) | (15, 26) |
| No change | 46 | 7.73 (18.42) | (3, 12) | 5.56 ( 19.42) | (1, 10) | 51 | -1.53 (19.5) | (-6, 3) | 2.18 ( 19.75) | (-2, 7) |
| Little better | 57 | 7.89 (19.47) | (4, 12) | 3.31 ( 17.97) | (-1, 7) | 68 | -7.68 (18.24) | (-11, -4) | 2.29 ( 22.92) | (-2, 7) |
| Much better | 49 | -2.27 (21.84) | (-8, 3) | 0.23 ( 18.63) | (-4, 5) | 59 | -13.40 (25.08) | (-19, -8) | -9.98 ( 21.11) | (-15, -5) |
| **Nausea and vomiting** | 255 |  |  |  |  | 255 |  |  |  |  |
| Much worse | 29 | 18.39 (26.48) | (10, 27) | 17.82 ( 25.17) | (10, 26) | 23 | -0.72 (39.72) | (-15, 13) | 10.14 ( 30.04) | (-1, 21) |
| Little worse | 74 | 8.78 (19.35) | (5, 13) | 8.33 ( 15.67) | (5, 11) | 53 | -3.14 (21.7) | (-8, 2) | 0.94 ( 17.73) | (-3, 5) |
| No change | 46 | -0.72 (12.65) | (-4, 2) | 0 ( 8.61) | (-2, 2) | 51 | -2.29 (10.01) | (-5, 0) | -1.31 ( 12.40) | (-4, 2) |
| Little better | 57 | 9.06 (21.38) | (4, 14) | 3.80 ( 17.82) | (0, 8) | 70 | -6.67 (23.98) | (-11, -2) | 0 ( 13.31) | (-3, 3) |
| Much better | 49 | -2.04 (15.07) | (-6, 2) | -2.72 ( 9.83) | (-5, 0) | 58 | -4.02 (11.81) | (-7, -1) | -5.17 ( 11.77) | (-8, -3) |
| **Pain** | 260 |  |  |  |  | 260 |  |  |  |  |
| Much worse | 32 | 24.48 (36.66) | (13, 35) | 25 ( 35.42) | (14, 36) | 23 | 13.8 (23.38) | (5, 22) | 26.09 ( 34.75) | (14, 39) |
| Little worse | 75 | 13.78 (24.87) | (9, 19) | 9.11 ( 23.14) | (5, 14) | 54 | 1.85 (22.35) | (-3, 7) | 17.28 ( 25.89) | (11, 23) |
| No change | 46 | 3.99 (14.57) | (0, 8) | 5.43 ( 17.23) | (1, 10) | 52 | 3.21 (14.02) | (0, 6) | 5.45 ( 17.06) | (1, 9) |
| Little better | 57 | 9.94 (20.62) | (5, 15) | 2.63 ( 21.08) | (-2, 7) | 70 | 2.14 (20.05) | (-2, 6) | 1.90 ( 22.97) | (-3, 6) |
| Much better | 50 | 8.33 (19.71) | (4, 13) | 4.67 ( 19.06) | (0, 9) | 61 | -12.3 (22.55) | (-17, -7) | -7.38 ( 20.30) | (-12, -3) |
| **Dyspnea** | 251 |  |  |  |  | 251 |  |  |  |  |
| Much worse | 30 | 17.78 (25.87) | (10, 26) | 22.22 ( 23.71) | (15, 30) | 23 | 4.35 (33.79) | (-8, 16) | 23.19 ( 30.87) | (12, 34) |
| Little worse | 73 | 8.68 (24.86) | (4, 14) | 10.96 ( 23.61) | (6, 16) | 52 | 3.85 (26.94) | (-2, 10) | 7.69 ( 18.22) | (3, 12) |
| No change | 45 | 0.74 (19.45) | (-4, 6) | 0.74 ( 18.10) | (-4, 5) | 51 | -0.65 (12.45) | (-4, 2) | 0 ( 11.55) | (-3, 3) |
| Little better | 55 | 1.21 (19.21) | (-3, 6) | 1.82 ( 16.25) | (-2, 5) | 67 | 3.48 (22.57) | (-1, 8) | 7.46 ( 23.08) | (3, 12) |
| Much better | 48 | -2.08 (21.09) | (-7, 3) | -2.78 ( 15.12) | (-6, 1) | 58 | -2.30 (23.25) | (-7, 3) | -1.15 ( 20.68) | (-6, 3) |
| **Insomnia** | 249 |  |  |  |  | 249 |  |  |  |  |
| Much worse | 30 | 15.56 (38.89) | (3, 28) | 28.89 ( 28.68) | (20, 38) | 23 | 4.35 (36.66) | (-9, 17) | 18.84 ( 39.98) | (5, 33) |
| Little worse | 69 | 5.80 (35.22) | (-1, 13) | 8.21 ( 28.24) | (3, 14) | 52 | 4.49 (30.98) | (-3, 12) | 15.38 ( 24.22) | (10, 21) |
| No change | 46 | -10.14 (35.04) | (-19, -1) | -2.90 ( 25.17) | (-9, 3) | 50 | -1.33 (21.25) | (-6, 4) | 2 ( 28.89) | (-5, 9) |
| Little better | 55 | -7.27 (34.95) | (-15, 1) | 0.61 ( 25.25) | (-5, 6) | 67 | 1 (30.13) | (-5, 7) | -0.50 ( 22.09) | (-5, 4) |
| Much better | 49 | -10.88 (36.25) | (-20, -2) | -3.4 ( 28.26) | (-10, 3) | 57 | -8.19 (26.19) | (-14, -2) | -11.7 ( 20.40) | (-16, -7) |
| **Appetite loss** | 249 |  |  |  |  | 249 |  |  |  |  |
| Much worse | 30 | 24.44 (43.71) | (11, 38) | 28.89 (33.6) | (18, 39) | 23 | -8.7(35.13) | (-21, 4) | 10.14 ( 32.47) | (-1, 22) |
| Little worse | 73 | 10.05 (28.16) | (5, 16) | 10.96 (27.25) | (6, 16) | 53 | -10.06(25.8) | (-16, -4) | 3.77 ( 20.32) | (-1, 8) |
| No change | 46 | -2.90 (20.88) | (-8, 2) | 1.45 (17.15) | (-3, 6) | 50 | -2.67(9.13) | (-5, -1) | -2 ( 12.44) | (-5, 1) |
| Little better | 54 | 1.85 (22.82) | (-3, 7) | 3.70 (15.41) | (0, 7) | 68 | -2.45(20.21) | (-7, 2) | -0.49 ( 14.67) | (-3, 2) |
| Much better | 46 | -8.70 (20.41) | (-14, -4) | -7.25 (18.48) | (-12, -3) | 55 | -4.85(14.93) | (-8, -1) | -7.88 ( 20.25) | (-12, -3) |
| **Constipation** | 247 |  |  |  |  | 247 |  |  |  |  |
| Much worse | 29 | 9.20 (40.72) | (-4, 22) | 17.24 ( 29.03) | (8, 26) | 21 | 4.76 (43.83) | (-12, 21) | 15.87 ( 41.66) | (0, 32) |
| Little worse | 68 | 15.20 (30.16) | (9, 21) | 10.29 ( 27.77) | (5, 16) | 48 | -1.39 (24.75) | (-7, 5) | 2.78 ( 21.56) | (-2, 8) |
| No change | 42 | 0 (19.48) | (-5, 5) | 2.38 ( 18.61) | (-2, 7) | 48 | -3.47 (12.38) | (-6, 0) | -1.39 ( 9.62) | (-4, 1) |
| Little better | 58 | 0 (26.49) | (-6, 6) | 0 ( 16.52) | (-4, 4) | 70 | -2.86 (30.95) | (-9, 3) | 0 (26.62) | (-5, 5) |
| Much better | 50 | 1.33 (23.29) | (-4, 7) | 1.33 ( 13.40) | (-2, 5) | 60 | -9.44 (27.51) | (-15, -4) | -4.44 ( 22.52) | (-9, 0) |
| **Diarrhea** | 248 |  |  |  |  | 248 |  |  |  |  |
| Much worse | 29 | 10.34 (25.36) | (2, 18) | 9.21 ( 23.40) | (2, 17) | 22 | -3.03 (30.7) | (-14, 8) | 1.52 ( 24.07) | (-7, 10) |
| Little worse | 71 | 0 (21.08) | (-4, 4) | 3.29 ( 18.07) | (0, 6) | 48 | 0.69 (14.57) | (-3, 4) | 4.17 ( 13.09) | (1, 7) |
| No change | 43 | -2.33 (15.25) | (-6, 2) | -0.78 ( 11.47) | (-4, 2) | 48 | -1.39 (9.62) | (-4, 1) | -2.08 ( 10.67) | (-5, 0) |
| Little better | 56 | -0.6 (21.55) | (-5, 4) | 2.38 ( 20.94) | (-2, 7) | 69 | -1.93 (17.97) | (-6, 2) | -1.93 ( 20.52) | (-6, 2) |
| Much better | 49 | -4.12 (17.52) | (-8, 0) | -0.70 ( 10.74) | (-3, 2) | 60 | -4.92 (17.04) | (-9, -1) | 2.19 ( 15.91) | (-8, -2) |
| **Financial difficulties** | 240 |  |  |  |  | 240 |  |  |  |  |
| Much worse | 26 | 14.10 (36.72) | (2, 26) | 11.54 ( 22.98) | (4, 19) | 21 | -3.17 (14.55) | (-9, 2) | 9.52 ( 28.17) | (-1, 20) |
| Little worse | 69 | 9.66 (22.94) | (5, 14) | 8.21 ( 22.44) | (4, 13) | 49 | -3.40 (20.69) | (-8, 2) | 4.76 ( 21.52) | (0, 1) |
| No change | 41 | 4.07 (13.32) | (1, 8) | 2.44 ( 8.79) | (0, 5) | 47 | 0 (0) | (NA) | -0.71 ( 4.86) | (-2, 0) |
| Little better | 58 | 1.15 (22.48) | (-4, 6) | 1.72 ( 15.82) | (-2, 5) | 66 | 1.52 (9.12) | (0, 3) | 0.51 ( 16) | (-3, 4) |
| Much better | 46 | 3.62 (21.35) | (-2, 9) | 2.17 ( 10.89) | (-1, 5) | 57 | 0.58 (14.76) | (-3, 4) | 1.17 ( 10.85) | (-1, 4) |

GHS: Global health status; Observed changes: Post-test – Pre-test; adjusted changes: Post-test – Then-test; SD: Standard Deviation; CI: Confidence Interval

**Table S4:** Observed and adjusted changes of the QLQ–BR23 questionnaire after three and six months

| **After 3 months** | | | | | | **After 6 months** | | | | |
| --- | --- | --- | --- | --- | --- | --- | --- | --- | --- | --- |
| **Between T0 and T1** | | | | | | **Between T1 and T2** | | | | |
|  |  | **Observed changes** | | **Adjusted changes** | |  | **Observed changes** | | **Adjusted changes** | |
| **QLQ-BR23** |  | **(post-test - pre-test)** | | **(post-test - then-test)** | |  | **(post-test - pre-test)** | | **(post-test - then-test)** | |
|  | **N** | **Mean (SD)** | **95% CI** | **Mean (SD)** | **95% CI** | **N** | **Mean (SD)** | **95% CI** | **Mean (SD)** | **95% CI** |
| **Body image** | 232 |  |  |  |  | 232 |  |  |  |  |
| Much worse | 28 | -47.22 (33.18) | (-58, -37) | -40.77 ( 34.65) | (-52, -30) | 21 | 1.59 (18.18) | (-5, 8) | -33.73 ( 35.89) | (-47, -20) |
| Little worse | 70 | -20.44 (27.41) | (-26, -15) | -15.40 ( 25.02) | (-20, -10) | 47 | 0.41 (16.58) | (-4, 4) | -10.76 ( 22.82) | (-16, -5) |
| No change | 41 | -7.32 (15.16) | (-11, -3) | -4.07 ( 15.60) | (-8, 0) | 46 | 2.72 (10.1) | (0, 5) | -0.91 ( 12.82) | (-4, 2) |
| Little better | 49 | -14.91 (24.41) | (-21, -9) | -4.93 ( 18.31) | (-9, -1) | 65 | 1.28 (22.93) | (-3, 6) | -1.45 ( 20.88) | (-6, 3) |
| Much better | 44 | -10.54 (28.43) | (-18, -3) | -2.53 ( 25.95) | (-9, 4) | 53 | 6.87 (20.87) | (2, 12) | 5.82 ( 21.26) | (1, 11) |
| **Sexual functioning** | 198 |  |  |  |  | 198 |  |  |  |  |
| Much worse | 26 | 12.82 (14.38) | (8, 18) | 18.59 ( 19.05) | (12, 25) | 26 | 7.89 (14.02) | (2, 13) | 24.56 ( 27.42) | (14, 35) |
| Little worse | 56 | 6.85 (20.79) | (2, 11) | 7.44 ( 21.06) | (3, 12) | 56 | -0.44 (12.55) | (-4, 3) | 10.53 ( 17.93) | (6, 15) |
| No change | 34 | 3.92 (22.87) | (-3, 11) | 5.88 ( 22.80) | (-1, 13) | 34 | 2.03 (16.33) | (-2, 6) | 2.44 ( 16.48) | (-2, 7) |
| Little better | 43 | 1.94 (15.94) | (-2, 6) | 0 ( 10.91) | (-3, 3) | 43 | -0.89 (18.1) | (-5, 3) | 0 ( 17.70) | (-4, 4) |
| Much better | 39 | -3.85 (21.45) | (-10, 2) | -4.27 ( 18.62) | (-9, 1) | 39 | -6.82 (14.52) | (-10, -3) | -5.68 (20.63) | (-11, 0) |
| **Sexual enjoyment** | 74 |  |  |  |  | 74 |  |  |  |  |
| Much worse | 8 | 4.17 (51.75) | (-31, 39) | 0 ( 43.64) | (-29, 29) | 8 | 8.33 (16.67) | (-11, 28) | 33.33 ( 27.22) | (1, 65) |
| Little worse | 23 | 14.49 (19.66) | (7, 22) | 11.59 ( 19.09) | (5, 18) | 23 | 12.50 (29.5) | (0, 25) | 14.58 ( 20.97) | (5, 24) |
| No change | 16 | 10.42 (33.82) | (-4, 25) | 4.17 ( 29.50) | (-9, 17) | 16 | 1.45 (21.27) | (-6, 9) | 2.90 ( 28.27) | (-7, 13) |
| Little better | 11 | 12.12 (22.47) | (0, 24) | 9.09 ( 21.56) | (-3, 21) | 11 | -2.08 (22.67) | (-12, 8) | 0 ( 21.08) | (-9, 9) |
| Much better | 16 | 2.08 (19.12) | (-6, 10) | -2.08 ( 8.33) | (-6, 2) | 16 | -6.67 (22.554) | (-17, 4) | -4.44 ( 24.77) | (-16, 7) |
| **Future perspectives** | 236 |  |  |  |  | 236 |  |  |  |  |
| Much worse | 31 | 3.23 (33.73) | (-7, 14) | -3.23 ( 40.69) | (-16, 9) | 31 | -3.03 (28.93) | (-14, 8) | -21.21 ( 40.6) | (-36, -6) |
| Little worse | 68 | 7.35 (32.5) | (1, 14) | -8.82 ( 30.81) | (-15, -3) | 68 | 3.47 (30.16) | (-4, 11) | -0.69 ( 24.30) | (-7, 5) |
| No change | 43 | 20.93 (32.55) | (13, 29) | 7.75 ( 26.06) | (1, 14) | 43 | 0 (23.57) | (-6, 6) | 4.76 ( 30.43) | (-3, 12) |
| Little better | 48 | 10.42 (30.1) | (3, 18) | 3.47 ( 24.06) | (-2, 9) | 48 | 1.06 (25.38) | (-4, 6) | 8.47 ( 23.92) | (3, 13) |
| Much better | 46 | 5.07 (33.68) | (-3, 13) | 12.32 ( 24.70) | (6, 18) | 46 | 11.73 (32.48) | (4, 19) | 15.43 ( 26.47) | (9, 21) |
| **Systemic therapy side effects** | 252 |  |  |  |  | 252 |  |  |  |  |
| Much worse | 32 | 26.93 (25.54) | (19, 35) | 26.54 ( 23.54) | (19, 34) | 32 | 2.76 (23.65) | (-6, 11) | 25.95 ( 26.03) | (17, 35) |
| Little worse | 72 | 18.83 (21.51) | (15, 23) | 19.79 ( 18.60) | (16, 23) | 72 | -3.49 (18.26) | (-8, 1) | 10.27 ( 18.90) | (6, 15) |
| No change | 45 | 4.41 (15.02) | (1, 8) | 3.84 ( 14.03) | (0, 7) | 45 | 0.23 (10.2) | (-2, 3) | 2.45 ( 7.89) | (1, 4) |
| Little better | 54 | 6.02 (13.64) | (3, 9) | 6.02 ( 12.35) | (3, 9) | 54 | -4.19 (15.12) | (-7, -1) | 3.94 ( 19.39) | (0, 8) |
| Much better | 49 | -2.01 (20.41) | (-7, 3) | -0.49 ( 15.93) | (-4, 3) | 49 | -5.83 (11.78) | (-8, -3) | -3.30 ( 13.01) | (-6, 0) |
| **Breast symptoms** | 212 |  |  |  |  | 212 |  |  |  |  |
| Much worse | 25 | 23.89 (32.72) | (13, 35) | 24.44 ( 29.32) | (14, 34) | 25 | 2.78 (20.33) | (-6, 12) | 13.33 ( 20.12) | (4, 22) |
| Little worse | 57 | 14.08 (27.69) | (8, 2) | 18.08 ( 22.21) | (13, 23) | 57 | -4.31 (16.81) | (-8, 0) | 6.44 ( 18.26) | (2, 11) |
| No change | 36 | 10.34 (20.43) | (5, 16) | 9.72 ( 21.59) | (4, 16) | 36 | -3.57 (13.35) | (-7, 0) | 3.77 ( 14.28) | (0, 7) |
| Little better | 53 | 16.61 (21.1) | (12, 21) | 7.13 ( 18.87) | (3, 11) | 53 | -2.21 (20.17) | (-7, 2) | 3.63 ( 20.65) | (-1, 8) |
| Much better | 41 | 9.55 (24.41) | (3, 16) | 3.05 ( 18.04) | (-2, 8) | 41 | -10.71 (21.13) | (-16, -6) | -0.62 ( 16.04) | (-4, 3) |
| **Arm symptoms** | 235 |  |  |  |  | 235 |  |  |  |  |
| Much worse | 29 | 11.88 (19) | (6, 18) | 9.39 ( 25.94) | (1, 18) | 29 | 10.49 (21.04) | (2, 19) | 16.67 ( 23.57) | (7, 26) |
| Little worse | 67 | 7.88 (17.6) | (4, 11) | 8.54 ( 19.28) | (5, 12) | 67 | 0.58 (18.27) | (-4, 5) | 10.76 ( 19.53) | (6, 15) |
| No change | 41 | 3.39 (13.54) | (0, 7) | 4.74 ( 14.14) | (1, 8) | 41 | 1.89 (13.82) | (-1, 5) | 5.89 ( 14.65) | (2, 9) |
| Little better | 55 | 4.55 (17.51) | (1, 8) | 1.41 ( 13.83) | (-2, 5) | 55 | 0.69 (13.98) | (-2, 4) | 0.69 ( 16.93) | (-3, 4) |
| Much better | 43 | 10.34 (13.8) | (7, 14) | 3.10 ( 17.87) | (-1, 8) | 43 | -1.52 (14.12) | (-5, 2) | 0.20 ( 15.78) | (-3, 4) |

Observed changes: Post-test – Pre-test; adjusted changes: Post-test – Then-test

SD: Standard Deviation; CI: Confidence Interval
